# Contrasting expression patterns of coding and noncoding parts of the human genome upon oxidative stress

**DOI:** 10.1038/srep09737

**Published:** 2015-05-29

**Authors:** Antonis Giannakakis, Jingxian Zhang, Piroon Jenjaroenpun, Srikanth Nama, Norliyana Zainolabidin, Mei Yee Aau, Aliaksandr A. Yarmishyn, Candida Vaz, Anna V. Ivshina, Oleg V. Grinchuk, Mathijs Voorhoeve, Leah A. Vardy, Prabha Sampath, Vladimir A. Kuznetsov, Igor V. Kurochkin, Ernesto Guccione

**Affiliations:** 1 Division of Genome and Gene Expression Data analysis, Bioinformatics Institute A*STAR (Agency for Science, Technology and Research), 138671, Singapore; 2 Division of Cancer Genetics and Therapeutics, Institute of Molecular and Cell Biology, A*STAR (Agency for Science, Technology and Research), 138673, Singapore; 3 Department of Biochemistry, Yong Loo Lin School of Medicine, National University of Singapore, 119074, Singapore; 4 Institute of Medical Biology, A*STAR (Agency for Science, Technology and Research), 138673, Singapore; 5 Program in Cancer and Stem Cell Biology, Duke-NUS Graduate Medical School, 8 College Road, 169857, Singapore; 6 School of Biological Sciences, Nanyang Technological University, 138673, Singapore; 7 School of Computer Engineering, Nanyang Technological University, 639798, Singapore

## Abstract

Oxidative stress (OS) is caused by an imbalance between pro- and anti-oxidant reactions leading to accumulation of reactive oxygen species within cells. We here investigate the effect of OS on the transcriptome of human fibroblasts. OS causes a rapid and transient global induction of transcription characterized by pausing of RNA polymerase II (PolII) in both directions, at specific promoters, within 30 minutes of the OS response. In contrast to protein-coding genes, which are commonly down-regulated, this novel divergent, PolII pausing-phenomenon leads to the generation of thousands of long noncoding RNAs (lncRNAs) with promoter-associated antisense lncRNAs transcripts (si-paancRNAs) representing the major group of stress-induced transcripts. OS causes transient dynamics of si-lncRNAs in nucleus and cytosol, leading to their accumulation at polysomes, in contrast to mRNAs, which get depleted from polysomes. We propose that si-lncRNAs represent a novel component of the transcriptional stress that is known to determine the outcome of immediate-early and later cellular stress responses and we provide insights on the fate of those novel mature lncRNA transcripts by showing that their association with polysomal complexes is significantly increased in OS.

A large proportion of the human genome is pervasively transcribed producing a great variation of RNA species with little (or no) protein-coding ability. RNA transcripts longer than 200 nt are classified as long non-coding RNAs (lncRNAs)^1^,^2^. Compared with protein-coding genes, lncRNAs show little evolutionary conservation in sequence and have limited coding potential, as indicated by the lack of significant open reading frames (ORFs), typical initiation codon, 3’-untranslated regions (UTRs) and termination codon^3^. Moreover, lncRNAs expression is detected at significantly lower levels and is more tissue specific. However, lncRNAs share many features of mRNAs as they are frequently transcribed by RNA polymerase II (PolII) and are generally spliced, 5′-capped, and polyadenylated^4^,^5^. The identification of lncRNAs has changed profoundly the way a gene unit may be defined, but annotation for the majority of lncRNAs is still missing^6^.

Low level of pervasive transcription mainly derives from intergenic loci and functionality of these lncRNAs, if any, still needs to be established^6^,^7^. On the contrary, pervasive transcription occurs often at promoters and enhancer regions^8^. The majority of mammalian gene promoters are able to initiate transcription at both strands, a phenomenon known as divergent transcription^9^,^10^; an event which is often linked to concordant regulation of sense/antisense pairs^11^,^12^. Divergent transcripts, in yeast and mammals, are usually suppressed by various mechanisms, including nucleosome remodeling^13^, histone deacetylation^14^, gene loop formation^15^, rapid removal by RNA degradation^16^, ^17^, ^18^, ^19^ and early transcription termination due to high frequency of polyA sites (PAS) upstream of coding genes^19^,^20^. All these mechanisms ensure promoter directionality towards the “sense” direction of protein-coding genes^21^.

LncRNAs are often classified by their location and/or position (sense or antisense) in relation to the nearest coding gene or non-coding regulatory region. Studies so far have identified a wide range of RNA transcripts antisense to protein-coding genes, transcripts associated with promoters, enhancers or repetitive regions^22^. Furthermore, lncRNAs’ functions can be exerted either *in cis* or *in trans* or both. LncRNAs are expressed in tissue-, cell- and/or developmental-specific expression patterns^23^, ^24^, ^25^ that are abrogated in various types of cancer^26^. Hence, a comprehensive understanding of cellular processes in physio/pathological conditions will be incomplete without the analysis of contribution made by lncRNAs.

Adaptation to stress is an essential cellular process. Stress signals trigger a common intracellular signaling cascade, which leads to the activation of the stress-activated protein kinases (SAPKs). The SAPK pathways are activated by various types of physiological stresses and constitute an early and transient cellular stress response (CSR) that is a critical determinant of cell fate^27^. Activated SAPKs enter the nucleus and induce the transcription of a group of genes (the immediate-early genes, IEG) which in turn signal cell cycle arrest and cellular repair or apoptosis depending on the cell typeand the intensity of the stressor^28^. Cells that survive the outcome of CSR mount a second stress-type specific pathway, called cellular homeostasis response, until conditions change or return to physiological levels^29^.

In mammalian cells, lncRNAs are induced in various types of stress, including genotoxic stress^30^, oxidative/endoplasmic reticulum and hyper-osmotic stress^31^,^32^ and are involved in the heat shock response^33^, DNA damage response^34^ and hypoxia^35^, ^36^, ^37^. In yeast, several studies have also started to shed some light on the complexity of the stress-responsive transcriptome under several conditions such as growth in different fermentable carbon sources^17^, diauxic shift^38^, in response to oxidative stress in fission yeast^39^ and upon osmostress via Hog1^40^ which is the yeast protein homologue of the SAPK p38. Nevertheless, there is no reported study to date examining, at a genome-wide level, the spatial and temporal sub-cellular re-distribution of lncRNAs at the onset of CSR in mammalian cells.

In response to OS, mammalian cells exhibit a rapid and tightly regulated signaling cascade that results mainly in the induction of the transcription of protein-coding genes involved in the regulation of the cellular redox-state^41^. Even though transcription might play a central role in the early cellular stress response, the majority of the oxidant-triggered gene expression patterns are regulated by RNA turnover and translation^42^. In this study, we characterized the impact of OS at the transcriptional and post-transcriptional level at the coding and non-coding regions of human lung and skin fibroblasts. We found that while genes known to be involved in the OS response were up-regulated (e.g. immediate-early genes), the vast majority of the protein-coding transcripts were down-regulated and translationally arrested upon H_2_O_2_ treatment. In contrast, thousands of lncRNA transcripts, pervasively transcribed from previously uncharacterized genomic loci, were transiently induced by OS and associated with polysomes. We classified and characterized the lncRNAs in these loci based on their genomic annotation and strand orientation relative to their nearest gene: distal, overlapping, terminal-associated or promoter-associated. Among these, si-paancRNAs were transcribed in antisense direction upstream to protein-coding genes and showed, in terms of percentage in their class, the biggest transcriptional up-regulation, as well as the strongest association with light and heavy polysomes, in response to OS. We demonstrate that cellular OS response is mediated by PolII, from strong (promoters) and week (distal genomic regions) bidirectional sites, enriched for specific transcription factor-binding sites (ZFP161, ZFX, MEF2A and divergent-FOS motives). The transcriptional induction of paancRNAs was reversed by removal of the stimulus (H_2_O_2_) and abolished by pre-treatment with N-acetyl-cysteine (NAC), a ROS scavenger.

## Results

### Oxidative stress-induced changes in the cellular transcriptome

**Figure 1:**
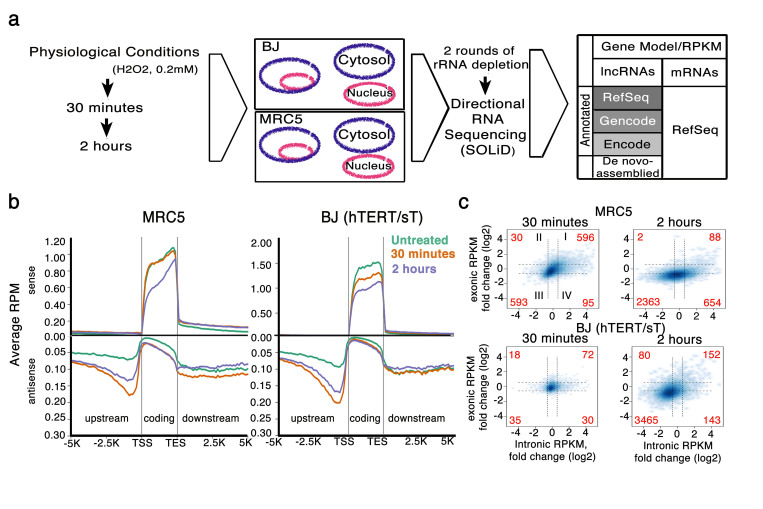
Analysis of the genomic response of fibroblast cell lines to oxidative stress (**a**) Methodology and RNA-seq pipeline (**b**) Distribution of the nuclear reads inside protein-coding genes, as well as in all strands upstream of their TSS (Transcription Start Sites) and downstream of their TTS (Transcription Termination Sites), in MRC5 and BJ cells that were either untreated, or treated with 0.2 mM H_2_O_2_.for 30 min or 2 hrs. Stress-induced antisense transcription upstream of protein-coding genes showed a significant peak both in height (read density) and length (transcript size) (**c**) Relationship between changes in the reads per kilobase per million (RPKM) values of all introns of a given gene and in the corresponding mRNA expression levels (exonic RPKM) at 30 min/2hrs of OS in MRC5 and BJ cells. Number of genes that were altered (>1.5-fold change) in intronic and/or exonic RPKM values are shown in red.

To capture changes in RNA levels in response to acute and transient OS, MRC5 and BJ fibroblasts were treated with 0.2 mM H_2_O_2_ at an early (30 min) and a late (2hr) time-point. Following nuclear and cytosolic fractionation of treated as well as non-treated cells, total RNA was isolated and directional RNA-seq libraries were generated (Fig.1a). Using our computational model of *de novo* expressed gene assembly and our cut-off criteria of confident gene expression value (Reads Per Kilobase per Million (RPKM) treated/control > 1.5 fold changes with filtering by treated reads > 10 and treated RPKM > 1, see Methods for more details), we found two distinct RNA expression patterns for the coding and non-coding parts of the genome across time and in both cell lines. Apart from a subset of Refseq/Genecode protein-coding genes that were sharply up-regulated, the majority of the differential expressed genes were down-regulated both in the nucleus (Fig.1b) and the cytoplasm (Fig.S1). In contrast, non-coding genomic regions showed increased transcription activity in the nucleus of both cell lines and time-points compared to non-treated cells (Fig.1b).

Gene ontology (GO) analysis showed that the sub-set of coding genes that are transcriptionally up-regulated in the nuclear and cytosolic fractions, in both BJ and MRC5 cells at 30 min and 2 hr following OS are involved in the cellular stress response and regulation of transcription (Suppl.TableS1, TableS2), while the down-regulated genes upon OS are involved in stress adaptation (Suppl.TableS1, TableS2).

Importantly, while the RPKM levels on exons are down-regulated after 30 min and 2 hr of OS, there was a general up-regulation of reads mapping within the introns of protein-coding genes. We calculated RPKM levels of reads mapping on all introns for each protein-coding gene and plotted them against the abundance of their mRNA levels (RPKM of reads mapping on exons). We found that OS results in an up-regulation (>1.5 fold-change) of intronic RPKM levels^43^ (quadrants I, IV Fig.1c) that is pronounced after 2 hr and more prevalent in MRC5 cells.

### RNA polymerase II accumulates transiently on both sides of active promoters upon stress

**Figure 2:**
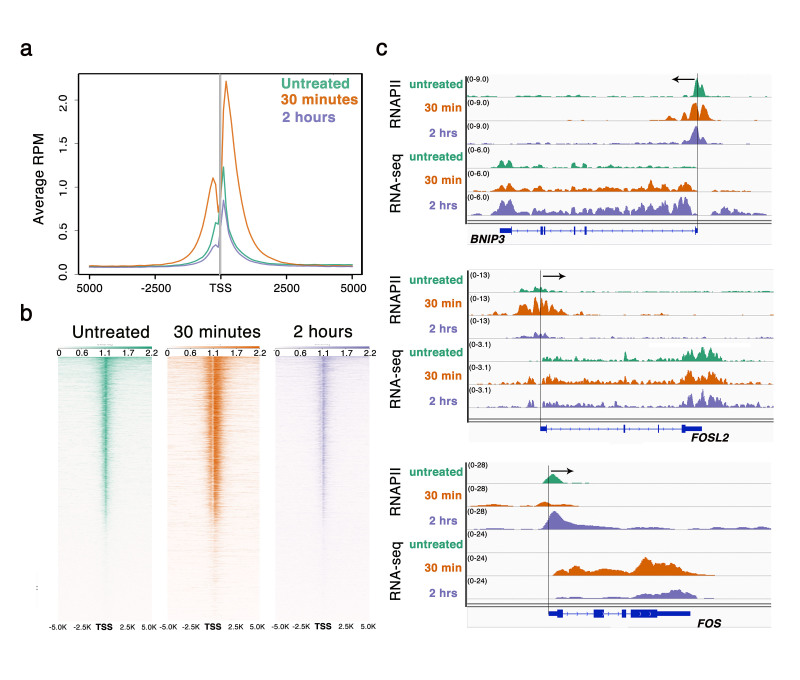
Chip-Seq binding distribution of RNA polymerase II in oxidative stress (**a**) Spatial distribution and (**b**) Heatmap representation of the distance of PolII binding around the TSS of all protein-coding genes in MRC5 cells treated with H_2_O_2_ (0.2 mM) for 30 min or 2 hrs, compared to untreated cells (**c**) Examples of PolII binding and RNA-seq read-distribution at loci of protein-coding genes.

Given the large changes observed in the transcriptome of both coding and non-coding parts of the genome, we decided to profile PolII occupancy of MRC5 in the same OS conditions. PolII transiently but significantly accumulates at promoters of protein-coding genes 30 min post H_2_O_2_ treatment. Interestingly, we observed accumulation of PolII at approximately 1 kb on both sides of the TSS (Fig.2a). At 30 min of OS, PolII not only accumulates at the TSS of genes with pre-loaded PolII, but is also released from the transcription start sites (TSSs) that were occupied before OS (Fig.2b). At 2 hr of OS, PolII genome-wide profiles return to the levels observed in untreated cells (Fig.2a-b). As examples, we present the up-regulated gene *BNIP3*, that exhibited a significant up-regulation of a transcript on the opposite strand and upstream of its TSS. (Figure.2c, top panel); and *FOSL2* (Fig.2c, middle panel), which is down-regulated at 2 hr upon OS. The promoter of *FOSL2* showed also a significant up-regulation of transcription on the opposite strand and direction. Additionally, the above cases exemplify the increase in the number of reads mapping within the introns of both genes in response to OS. It is important to stress that this bidirectional burst of transcription upon OS is occurring only at defined genomic loci. For example, the well-known immediately-early gene *FOS,* does not show upstream antisense transcription (Fig. 2c, lower panel). Before we could address the question of which promoters predominantly show upstream antisense direction, we had to establish which genomic loci are selectively transcribed during OS.

### Classification of stress-induced lncRNAs

**Figure 3:**
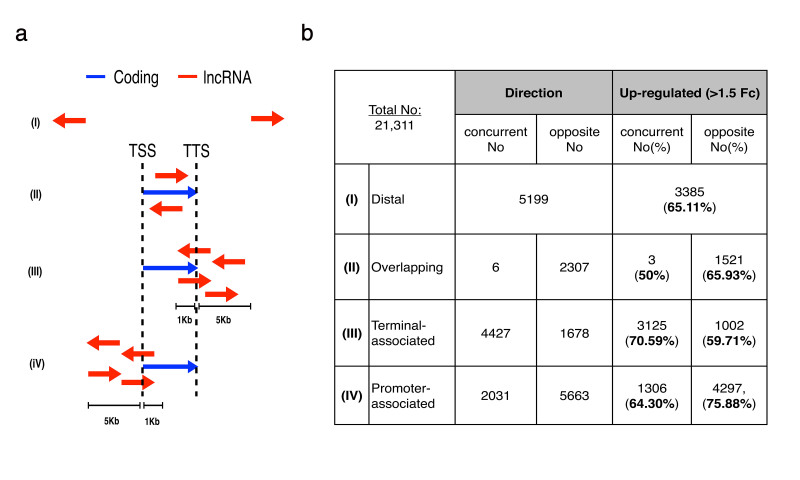
LncRNA classes identified in oxidative stress. (**a**) Cartoon representation of the distance and location of each lncRNA class in relationship to the nearest coding gene. The lncRNA transcripts (in red) could be in distal regions 5 kb away of any coding gene (in blue), overlap completely with coding genes in both directions (sense/antisense), located downstream (1 kb < TTS < 5 kb) or upstream (5 kb < TSS < 1 kb) in both strands (concurrent/opposite) in relation to the nearest coding gene (**b**) Table depicting the number of concurrent and opposite lncRNAs identified in each class and the percentage that was found to be up-regulated in either MRC5 or BJ cells at 30 minutes or 2 hours of OS.

We classified non-coding transcripts generated during OS by employing a two-step strategy. Firstly, we built our *de novo* assembly gene model (see Methods) for long non-coding RNA genes (lncRNAs, >200 nt in length), assigning the reads to publically annotated lncRNAs (RefSeq and Genecode,) or to unannotated ones (*de novo* assembled Cufflink transcripts) (Fig.1a). Secondly, all identified lncRNAs transcripts were grouped based on their strand location and position relative to the nearest RefSeq annotated protein-coding gene. This computational strategy resulted in the prediction of four classes of oxidative stress-induced lncRNAs (Fig.3a) as follows: (I) lncRNAs that are not in the proximity of any annotated gene (distal lncRNAs: dncRNAs), (II) lncRNAs overlapping protein-coding genes (concurrent and opposite), (III) lncRNAs associated with the transcription termination sites (TES) (concurrent and opposite) and (IV) promoter-associated lncRNAs (concurrent and opposite) (Suppl.TableS3).

In total, 21,311 lncRNAs were detected at significant levels (>10 reads, and >1RPKM) out of which 14,639 (68.7%) were up-regulated more than 1.5-fold in at least one time-point and one cell line in the nuclear fraction. Three lncRNAs groups, based one absolute transcript numbers detected showed up as the most prevalent classes. These were: dncRNAs (N = 5199, 65.1% up-regulated); antisense overlapping lncRNAs (N = 2307, 65.9% up-regulated); terminal-associated lncRNAs transcribed at the same direction as their protein-coding genes pairs (concurrent) (N = 4427, 70.7% up-regulated); and promoter-associated antisense lncRNAs (paancRNAs) (N = 5663, 75.9% up-regulated) which, interestingly, showed the highest rate of inducible transcripts expressed as percentage of their corresponding class size. This analysis revealed a strong contrast between a general up-regulation of the levels of pervasive transcription in the non-coding portion of the genome and down-regulation of the bulk of the protein-coding part in response to OS (Fig.3b).

### Distinctive length of stress-induced transcripts

**Figure 4:**
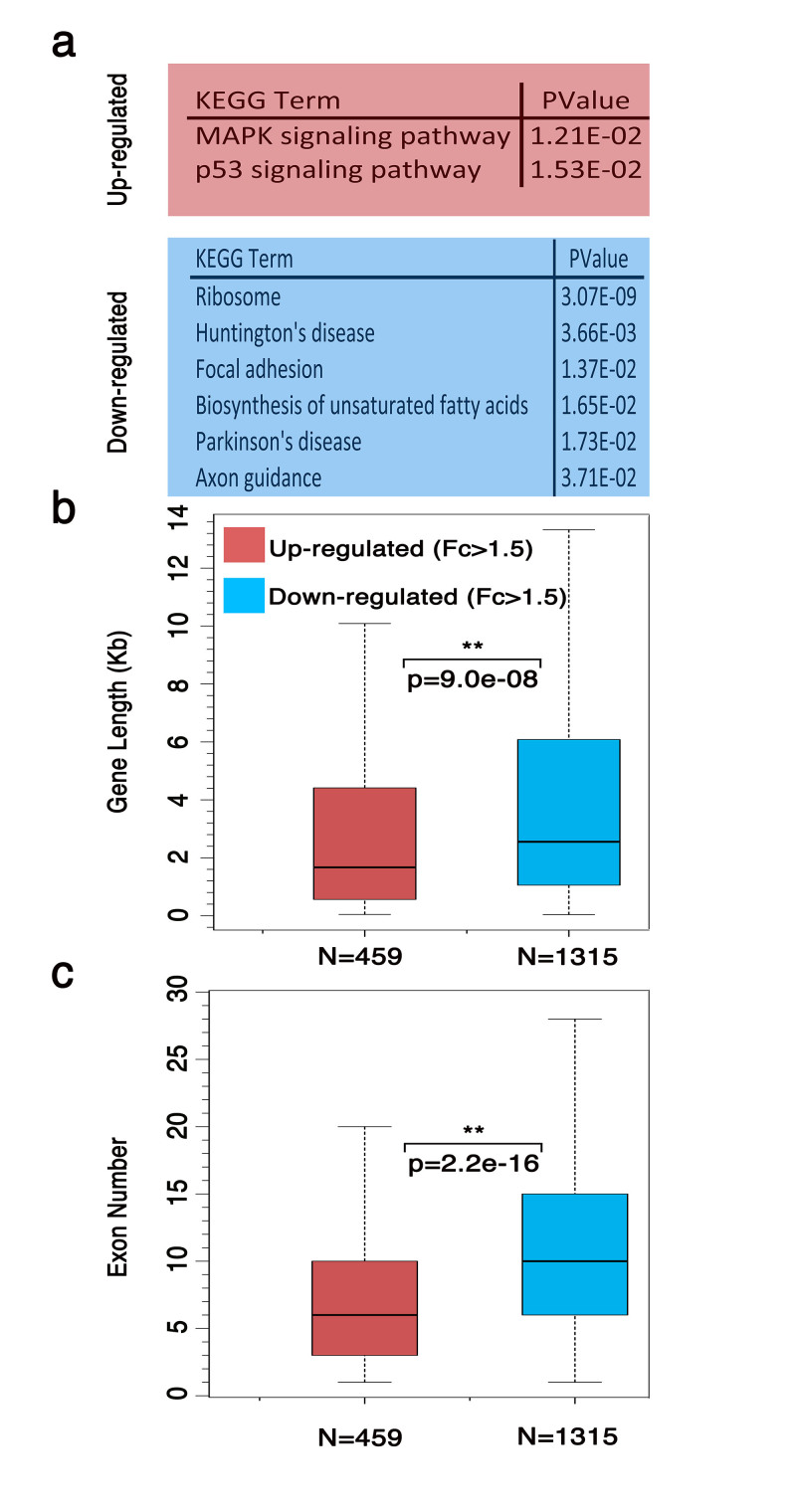
Gene structure analysis of differential expressed coding genes upon OS. (**a**) KEGG pathways associated with up-regulated and down-regulated coding genes in OS. Box-plot analysis of the distribution of the gene length (**b**) and exon number (**c**) of up-regulated (in red) and down-regulated (in blue) coding genes in the nucleus of both cell lines (MRC5 and BJs) and at both OS time-points (Mann–Whitney–Wilcoxon).

As expected, we identified that the subset of coding genes commonly up-regulated in 30 min and 2 hr in both cell lines are involved in MAPK signaling (Fig.4a) and have the typical gene structure of immediate early response genes^44^: i.e. they are on average shorter (Fig.4b) and have fewer number of exons than coding genes which are down-regulated under the same conditions (Fig.4c). The coding genes down-regulated in the nucleus (which represent the majority of the differentially expressed genes) following OS, showed an enrichment for GO terms that are more relevant to homeostasis and adaptation to oxidative stress like ribosome, focal adhesion and metabolic activities (Fig.4a).

Interestingly, we found that stress-induced lncRNAs are similarly shorter in length (Fig. S2a, b, c) and the vast majority (~90%) are mono-exonic. In this respect, they resemble the structure of coding genes that are up-regulated under the same conditions. This observation suggests that a great number of stress-induced lncRNAs may belong to the same subset of genes responsible for driving the cellular stress response.

### Promoter-associated antisense lncRNAs (paancRNAs) are up-regulated upon oxidative stress

**Figure 5:**
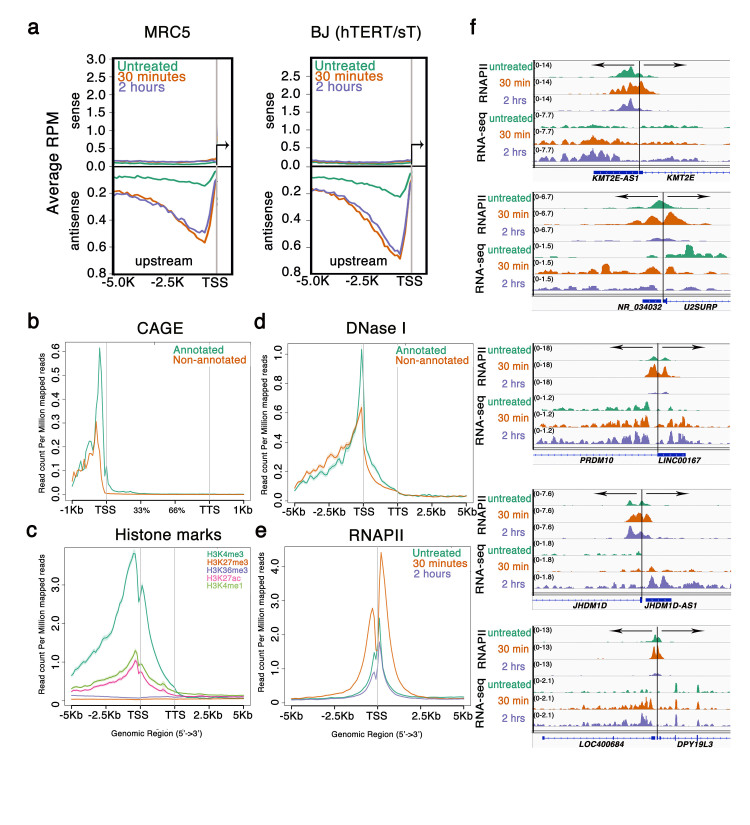
Characterization of si-paancRNAs (**a**) Density distribution analysis of RNA-seq reads upstream of the TSSs of the protein-coding partners of si-paancRNAs (**b**) overlap of si-paancRNAs with CAGE tags, (**c**) histone marks and (**d**) DNase I hypersensitivity sites (**e**) Distribution of PolII binding around the predicted TSS of paancRNAs in OS compared to untreated MRC5 cells (**f**) Examples of PolII binding and RNA-seq read distribution at si-paancRNAs loci.

Divergent transcription of si-paancRNAs, peaking between −0.5 kb to −1.5 kb upstream and in the opposite direction of the protein-coding genes, is the predominant source of stress-induced lncRNAs (Fig.3b, Fig.5a). The median length of the previously annotated si-paancRNAs (N = 1762) is 1405 bp, while that of the de-novo-annotated si-paancRNAs (N = 3091) is 478 bp (Fig.S2a).

All these paancRNAs potentially represent mature transcripts. Both long (annotated) and intermediate-sized (de-novo annotated) si-paancRNAs are enriched for CAGE tags, which define the 5’ termini of RNA transcripts, immediately upstream of their predicted gene starts (Fig.5b). Similar analysis using publicly available Chip-Seq libraries for histone modifications and DNase I Hypersensitive sites revealed that the vast majority of si-paancRNAs are enriched for H3K4me3 (Fig.5c) and DNase I accessible sites (Fig.5d) and to a minor extent for H3K4me1 and H3K27ac, while no si-paancRNAs overlap with sites enriched for the repressive H3K27me3 mark (Fig. 5c). Similarly to what was observed genome wide, the mRNA/si-paancRNA pairs display a significantly strong accumulation of PolII at 30 min, both towards the gene bodies of the RefSeq si-paancRNAs and their protein-coding counterparts (Fig.5e-f).

Representative examples highlight the fact that PolII accumulation at 30 min (validated by ChIP-qPCR, Fig.S3a) is accompanied by a burst of transcription (Fig.5f). The same result can also be visualized by plotting the PolII stalling index/ traveling ratio (PolII present at promoter/PolII present in the gene body) in MRC5 after 30 minutes of OS (Fig.S3b).

### Si-paancRNAs promoters are enriched for specific TF binding sites

**Figure 6:**
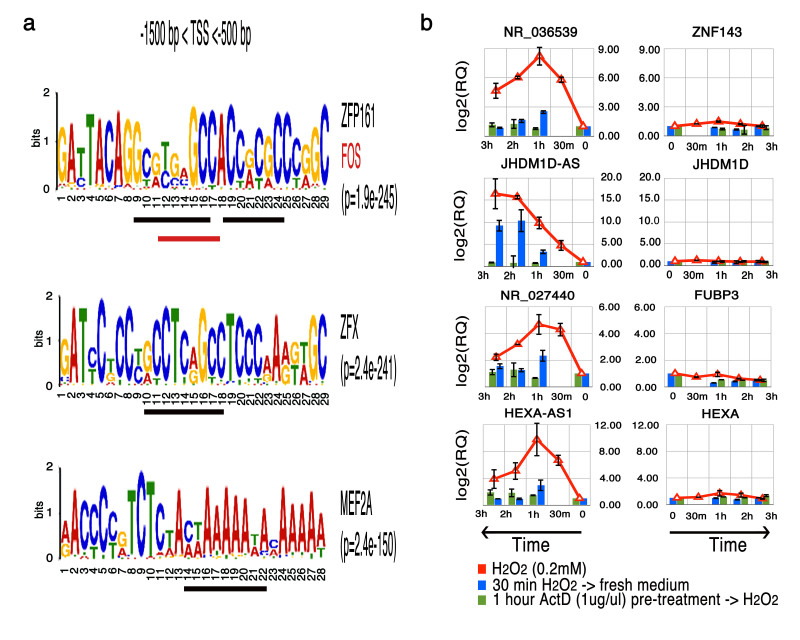
Si-paancRNAs promoters are enriched for specific TF binding sites and their expression is PolII dependent (**a**) *De-novo* motif analysis of the genomic region upstream of the TSS of the protein-coding partners of si-paancRNAs (−1500 bp < TSS < −500 bp). *Motif1*: 2080/3803 = 54.69%, *motif2*: 2262/3803 = 59.48%, *motif3*: 2258/3803 = 59.37% (**b**) Transcript abundance kinetics of si-paancRNAs in MRC5 cells treated with 0.2mM H_2_O_2_ (red line), depletion of H_2_O_2_ (blue bar) and following 1hr pre-treatment with Actinomycin D before exposure to 0.2mM H_2_O_2_ (green bar).

Motif analysis of a 1 kb genomic region 0.5 Kb upstream of the protein-coding gene pairs of paancRNAs (putative si-paancRNAs promoters) revealed a significant enrichment of three motifs (Fig. 6a). Overall, motif1 was detected 4410 times and on average, two times (4411/2080 promoters = 2.1) at each promoter region. Similar or greater co-occurrence frequencies were observed for motif2 (4789/2262 = 2.1) and motif3 (7953/2258 = 3.5). Strikingly, not only all the three motifs showed, individually, an increased co-occurrence frequency, but also 36.9% of the promoters of the coding pairs of si-paancRNAs analyzed (1404 of 3803 regions) were found to contain all three motifs simultaneously. Moreover, similar motives were also enriched at promoters of dncRNAs, implying that stress-induced lncRNAs (both dncRNAs and paancRNAs) are regulated by a common set of transcription factors (Fig.S4). The most significant, motif1 (29 nt) contained the binding sites of ZFP161 and a divergent FOS motif. ZFP161 is a transcription factor containing a ZiN/POZ domain. It can repress (e.g. c-myc, herpes simplex virus, thymidine kinase, β-actin) or activate (e.g. HIV-1, dopamine transporter) transcription, depending on the cell and gene context^45^. Interestingly, ZFP161 is postulated to be involved in the regulation of bi-directional transcription^46^ and we identify two putative ZFP161 binding sites, within the predicted long motif (Fig. 6a-top and Fig.S4-middle motif). One of the sites is overlapping the putative divergent binding sites of FOS, a well-known transcription factor involved in the OS response^47^.

To ensure a rigorous validation of the genome-wide computational analysis, we selected all RefSeq lncRNAs that were commonly up-regulated in both cell lines and at both time-points (30 min and 2 hr) for RT-qPCR quantification (Fig.S5a). We successfully validated all (24/24) lncRNAs tested. Fifteen (15), out of 24 lncRNAs tested belonged to what we have defined as si-paancRNAs (Fig.S5b). To understand if this genome-wide up-regulation of non-coding transcripts was due to PolII-mediated transcription or merely the result of stress-related defects in the exosome RNA degradation machinery we analyzed their transcript stability in H_2_O_2_-stressed MRC5 cells (for 30 min) in which we blocked transcription by addition of actinomycin D (ActD). Pre-treatment with ActD abolished the induction of si-paancRNAs, while it had no significant effects on their corresponding protein-coding pairs (Fig. 6b and Fig.S6a). We further examined whether transcriptional up-regulation of si-paancRNAs is dependent on the presence of reactive oxygen species (ROS). We found that pretreatment with *N*-acetyl-l-cysteine (NAC), a ROS scavenger abolished induction of si-paancRNAs (Fig. S6b).

### Distal long non-coding RNAs (dncRNAs) are up-regulated upon oxidative stress

To test whether this burst of bidirectional PolII accumulation was localized only at promoters we extended our analysis to dncRNAs. De-novo annotated dncRNAs are shorter than their RefSeq counterparts, with a median transcript size of 425 bp and 953 bp, respectively (Fig.S2b). Both annotated and de-novo assembled dncRNAs expression increases upon OS, and even at these distal sites we detect an accumulation of reads in the antisense direction (Fig.S7a). To further characterize OS-induced dncRNAs loci, we utilized publicly available datasets on DNase I hypersensitivity sites and CAGE-tags generated from BJ fibroblasts. Both annotated and de-novo assembled dncRNAs originate from DNaseI hypersensitive sites (markers of nucleosome depleted regions) (Fig.S7b). However, enrichment for CAGE tags at their TSS was weak (Fig.S7c). Next, we assessed whether nucleosomes around the TSSs of dncRNAs were enriched for H3K4me3 (a marker for active promoters), H3K4me1 (typically present at enhancers) and H3K27ac (marker for active enhancers). To our surprise, the majority of dncRNAs didn’t show detectable levels of H3K4me3, H3K4me1 and H3K27ac (Fig.S7d), while a small proportion (22.6%) were associated with H3K4me3, probably representing un-annotated promoters, and 13.8% with both H3K4me1 and H3K27ac, underscoring the presence of active enhancers. Nonetheless, we validated by qPCR the over-expression of five (5) dncRNAs (Fig.S7e) and we show that, in response to OS, PolII accumulates transiently at regions were it was already present prior to OS and frequently elongates bi-directionally (Fig. S7f, S7g-middle and lower panel), similarly to what it was observed at promoters of protein-coding genes.

### Si-paancRNAs associate with the translational machinery in oxidative stress response

**Figure 7:**
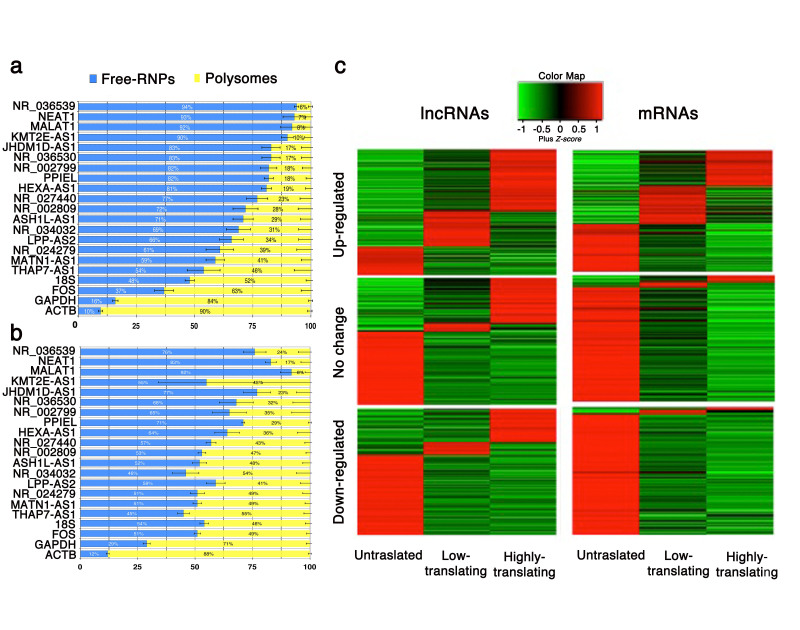
Si-paancRNAs associate with polysomal complexes upon oxidative stress. Transcript enrichment in Free-RNP fractions (un-translated pool) versus polysomal complexes (translated pool) was analyzed by RT-qPCR and calculated as percentage to the total transcript levels for each RefSeq si-paancRNA in untreated (**a**) and treated MRC5 cells with H_2_O_2_ for 2 hrs (**b**) The experiment was performed in three independent biological triplicates. MALAT1/NEAT1 (lncRNAs), GAPDH/ACTB/FOS/(protein-coding genes) and 18S (rRNA) were used as controls (*denotes t-testp-value > 0.05) (**c**) Heatmap display of custom microarray analysis on transcriptionally up-regulated (Fc > 1.5, in whole-cell lysates) Gencode (v15) transcript levels (fold-change) within three polysome pools (single-RNPs, light and heavy polysome complexes) in MRC5 cells treated with H_2_O_2_ for 2 hrs compared to untreated cells. Stress-Induced lncRNAs (Fc > 1.5) associate better with light and heavy polysomes compared to stress-induced mRNAs. Expression levels (up-regulated, no change or down-regulated) are indicated on the left of each panel for both lncRNAs and coding mRNAs.

An imperative for the functional analysis of lncRNAs is the high-confidence in their annotation as truly no-protein coding. The operational distinction of a transcript’s protein-coding ability relies on its ability to associate either with free ribonucleoproteins (free-RNPs) or with polysomes. By performing polysome profiling combined with RT-qPCR analysis we found that different si-paancRNAs have different transcript enrichment patterns in the translated and untranslated fraction pools (Fig. 7a). Reassuringly, when sorted based on their enrichment in the translated polysome fraction, they showed a significant correlation with the corresponding coding bias index (CBI) that was estimated for the longest predicted open reading frame (ORF) of each lncRNA (Fig.S8a). Positive CBI was observed for RefSeq si-paancRNAs with enrichment <70% in the small RNPs fractions. NEAT1 and MALAT, two well-characterized intergenic lncRNAs, were used as controls for the untranslated fraction, while GAPDH, ACTB and FOS mRNAs, for the translated fraction. Si-paancRNAs scored in the middle, showing an ability to associate with both small RNPs and with larger polysome protein complexes, with the potential to be translated into small novel polypeptides^48^. Even though si-paancRNAs show a significant localization to the nucleus, all of them could be detected at moderate levels in small, medium or high ribosomal fractions isolated from untreated cells.

Given the association of some si-paancRNAs with the polysome fractions in non-stressed conditions, we investigated whether their distribution on the ribosomal complexes would be perturbed upon stress. Remarkably, the RefSeq paancRNAs originally identified in this study showed, in bulk, an increased association with polysomes at 2 hours of OS (Fig. 7b). Interestingly, the behavior of RefSeq paancRNAs was strikingly different from the overall polysome distribution in OS, (Fig.S8b), which represents a typical translational arrest in stress conditions^49^ (see also FOS, GAPDH, ACTB mRNAs in Fig. 7b).

Having demonstrated increased association of selected si-paancRNAs with polysomes upon OS, we were interested to examine whether other si-lncRNAs behaved similarly at a global scale. We thus decided to utilize a custom microarray capable of quantifying 22,001 lncRNA transcripts (GENCODE v15 annotation). The array also targeted 17,535 randomly selected mRNAs (see Methods). As the number of ribosomes per transcript indicates translational efficiency of RNA, we sought to obtain single ribosome resolution profiles (see Methods). Three pools derived from sucrose gradient fractionation were prepared for quantitative microarray analysis: untranslated (free RNPs and 80S), low-translating (two-four ribosomes), and highly-translating (>five ribosomes). Upon OS the majority of protein-coding transcripts moved away from the light and heavy polysome fractions. The exception consisted of a small fraction of coding mRNAs, which needs to be translated as part of the stress response (Fig. 7c). In stark contrast, the majority of lncRNAs shifted toward the heavier polysome fractions upon OS, with si-paancRNAs showing the highest frequency in all the three fraction types (Fig.S8c), validating our initial polysome profiling (Fig. 7b). The microarray results were confirmed, for representative transcripts, by qRT-PCR (Fig. S8d).

These results revealed contrasting response of mRNAs and lncRNAs in terms of their association with the translational machinery upon OS.

## Discussion

Several studies in yeast have reported that the environmental core stress response is a two-step process that involves first an acute and transient burst in the transcript levels and a subsequent down-regulation of the protein levels before cells acclimate^50^, ^51^, ^52^. In mammalian cells, the core stress response seems to include only a small set of mRNA genes, which is enriched for regulatory functions (e.g. immediate-early genes). In the current study, we show that, similarly to yeast, lncRNAs show contrasting expression patterns compared to mRNAs in normal fibroblast cells exposed to acute OS and that indeed the core stress pathway in human cells triggers a far greater transcriptional response than previously anticipated. the response to oxidative stress of mammalian cells is a rapid and global induction of transcription characterized by a sharp pausing of polII in both directions (sense/antisense) within 30 minutes post OS response. This novel bidirectional transient pausing-phenomenon is likely causing post-transcriptional processing defects that lead to the down-regulation of the mRNA levels of most coding genes , except of genes involved in the stress core response that are structurally shorter in length and have significantly less number of exons. Among the identified si-lncRNAs, we show that paancRNAs and to a smaller extent, dncRNAs represent capped and poly-adenylated transcripts originating from open chromatin regions. Terminal-associated si-lncRNAs, in the same direction of their coding partner, might be generated due to processing defects related to transcriptional termination, since we were not able to map any significant polII peaks at their annotated TSSs.

Additionally, we show that OS promotes the transcription of si-paancRNAs, but not of their protein-coding partners, which are longer and require additional signals for productive elongation. Si-paancRNAs possess typical features of mRNAs and upon OS, they are induced and enriched to sub-polysomal and polysomal fractions, suggesting that they are indeed passing quality control, while escaping nuclear degradation, representing a potential depository of RNA transcripts that have gone one step further on the path to function. The functional significance of stress-induced lncRNAs will need to be further elucidated and at least three possible roles for these transcripts come to mind: (**1**) to participate in *de novo* gene origin; (**2**) to be involved in the core environmental stress response of cells by recruiting transcription factors or RNA-biding proteins involved in the DNA damage response (DDR) or heterochromatin formation (**3**) to associate with ribosomal proteins forming stress-specific RNPs as part of the protection/adaptation to OS.

### Oxidative stress may drive de-novo gene origin

Protein-coding gene bodies are characterized by a low presence of A-rich (AAUAAA or similar) poly(A) signal (PAS) motives^53^, and a high presence of G/T rich U1 snRNP-binding sites, which allow the recognition of the 5’-donor splicing site. This strong U1-PAS axis promotes the expression of full-length mRNAs by blocking premature termination, cleavage and polyadenylation^20^. Based on a recent model of gene creation proposed by Wu and Sharp, the act of antisense transcription, which is widespread in germ cells^21^ strengthens the U1-PAS axis in the upstream antisense region (si-paancRNAs) or distally at regulatory regions (dncRNAs). A recent study demonstrates that ROS are essential for spermatogonia proliferation and self-renewal^54^, raising the intriguing possibility that ROS might also be propelling de novo gene origin in the germ line.

### Stress-induced lncRNAs are integral part of the core transcriptional response to environmental stress

It is well documented that the cellular response to stress is a highly conserved pathway that involves the transcriptional induction of a common set of immediate early genes regardless of the stress type. In accordance with a previous study^44^ we observed that protein-coding genes up-regulated by OS and enriched for the MAPK signaling stress response are shorter in gene length and have less number of exons, compared to genes that are down-regulated and enriched with biological processes involved in the adaptation to stress. We report that si-paancRNAs and dncRNAs are akin to typical immediate early genes, as they share similar genomic features of immediate-early genes that facilitate rapid induction. Thus, si-paancRNAs and dncRNAs (together with immediate-early genes) might orchestrate the early stress core response by targeting transcriptional activators or repressors to specific promoters.

Stress-induced paancRNAs originate from promoter regions that are enriched for long motifs that include putative binding sites for FOS, ZFP161, MEF2A and ZFX. FOS belongs to a group of immediate early genes that are rapidly and transiently activated upon a variety of environmental stress conditions. In our experiments, FOS mRNA levels were induced more than 20-fold within 30 min of fibroblasts treatment with H_2_O_2_. However, ZFP161, MEF2A and ZFX did not show up-regulated transcript levels. The activity of transcription factors may be regulated by nuclear localization, post-translational modifications etc. MEF2A is a substrate for ERK5^55^ and p38^56^, both of which are rapidly activated by H_2_O_2_ in a variety of human cells. Interestingly, activation of these MAPK kinases is also found to markedly increase the transactivation activity and mRNA half-life of FOS^57^,^58^ raising the possibility of a coordinated regulation of FOS and MEF2A. Additionally, MEF2A was shown to be involved in the up-regulation of KLF2, a transcriptional regulator of detoxifying enzymes, to regulate ROS levels in cells^59^. Remarkably, all three motifs identified at si-paancRNA promoters were frequently found in several copies, and showed increased co-occurrence frequency; potentially implying that activation of paancRNA genes requires the cooperative action of several different transcription factors. This peculiar promoter structure might be essential to ensure a rapid and strong induction of paancRNAs transcription upon OS. The presence of the binding site for ZFP161 might strengthen the bidirectional nature of these promoters since ZFP161 was demonstrated to be involved in bi-directional control of gene expression^46^.

The fact that si-paancRNAs are transcribed from the promoters of genes involved in the oxidative stress response makes this co-regulation likely to be functional. Indeed promoters of both si-paancRNAs and dncRNAs, themselves contain a divergent binding motif for FOS (GTGAGCCA as opposed to the canonical GTGAxTCA), one of the most well characterized immediate-early genes. Follow up experiments will be necessary to address the functionality of these binding sites. FOS is highly expressed in different cancers and is a prognostic marker for cancer progression. Therefore high expression levels of si-paancRNAs and dncRNAs are expected in various cancer cells. Cellular response to OS thus might offer a valuable model to uncover lncRNAs functionally implicated in carcinogenesis.

### Si-lncRNAs associate with the translational machinery and may facilitate cellular adaptation to stress

The adaptation of mammalian cells to OS involves global translational arrest. While the majority of mRNAs shifted upon OS to a lighter, non-translating fraction, lncRNAs displayed strikingly opposite behavior. The majority of stress-induced lncRNAs, especially of si-paancRNAs, accumulated on polysomes. The biological relevance of stress-induced redistribution of lncRNAs to the translational machinery is unclear. We could envision multiple ways in which association of lncRNAs with polysomes may contribute to integrated stress response. Si-lncRNAs may increase the translation of a subset of mRNAs upon stress. Although there is global translational arrest following oxidative stress, a defined group of mRNAs is highly translated and this maybe facilitated by chaperons that favor translation of stress-specific RNP complexes. Recent data show that some lncRNAs could enhance the translation of specific protein-coding transcripts through sequence-specific pairing^60^. Furthermore, the cap-dependent 5’-terminal initiation is used commonly in eukaryotic mRNA translation and it involves a tightly controlled multi-protein mRNA cap-bound eIF4F complex^61^. The eIF4F complexes are present in a limited amount and in this situation cytoplasmic mRNAs compete for their binding. It is plausible that lncRNAs massively induced by OS effectively compete with cellular mRNAs for binding to the eIF4F complexes and in this way facilitate their movement away from polysomes resulting in translational repression. This might be especially true for si-paancRNAs that are likely capped as their predicted TSSs are enriched for CAGE tags. We cannot, however, exclude the very attractive hypothesis that polysome-bound lncRNAs are translated and, thus, have protein coding potential. Recent large-scale mass-spectrometry experiments involving various human tissues and cell lines revealed 430 high-quality peptides from 404 lncRNAs^62^. These peptides may be a result of spurious translation of lncRNAs and their biological significance is not clear at present. However, at least some polypeptides encoded by lncRNAs are functional. For example, human transcript AK092578, annotated as lncRNA, is translated to produce a secreted peptide hormone of 32 amino acids essential for heart development^63^. Our future studies will be focused on testing whether stress-induced redistribution of si-lncRNAs to polysomes results in production of translated products and on possible role of the produced polypeptides in providing increased tolerance to oxidative stress.

## Methods

### Cell culture

MRC5 and BJ fibroblasts were cultured in 5% CO_2_ atmosphere, at 37 °C in DMEM medium supplemented with 10% fetal bovine serum (FBS) and penicillin/streptomycin. The BJ cells used in this study were semi-immortalized by stably expressing hTERT and SV40 small T. Both cell lines were seeded at sub-confluent numbers (80–90%) and treated the next day for 30 min or 2 hr with 0.2 mM H_2_O_2_.

### Cellular Fractionation

For the preparation of nuclear and cytosolic fractions, a modified version of the Dignam protocol^64^ was used. Briefly, 10^7^ cells were collected by scrapping/centrifugation and washed twice in ice cold PBS. Cell pellets were re-suspended into 250 μl of ice-cold Homogenization Buffer (100 μM Tris-Cl pH 7.5, 15 mM NaCl, 60 mM KCl, 7.35 ml 150 mM sucrose, 0.05% NP-40, 1 mM DTT, 10 mM Vanadylribonucleoside complex (VRC, New England Biolabs) and proteinase inhibitors). One fifth of the cell lysate (50 μl) was transferred to a new tube and used to isolate total RNA from whole cell extracts while the remaining was stratified onto an equal-volume of sucrose pad (100 μM Tris-Cl pH 7.5, 15 mM NaCl, 60 mM KCl, 300 mM sucrose, 1 mM DTT, 10 mM VRC, proteinase inhibitors). Nuclear and cytosolic fractions were separated by centrifugation at 900 rpm for 10 min at 4 **°**C (program without brake). Each supernatant (cytosolic fraction) was transferred to a new tube while nuclei pellet were re-suspended in 250 μl of Wash Buffer (100 μM Tris-Cl pH 7.5, 15 mM NaCl, 60 mM KCl, 1 mM DTT, 10 mM VRC, proteinase inhibitors) followed by centrifugation at 14,000 rpm at 4 **°**C for 30 min to get rid of cytoplasmic residue. One ml of TRIzol was added directly to whole cell extracts, nuclei pellet and cytosolic fractions.

### Total RNA Isolation

Total RNA was isolated from 1–1.5 × 10^6^ cultured cells with TRIzol reagent (Invitrogen). For RNA-seq the aqueous phase containing total RNA from whole cell extracts, nuclei and cytosol was purified using a modified protocol of the Purelink RNA mini kit (Life technologies). RNA samples from three biological replicates for each timepoint and fraction were combined in equimolar amounts and 5 μg of these RNA samples were then treated twice with Ribominus RNA removal kit (Life Technologies) and was concentrated using the Ribominus concentration module (Life Technologies). RNA quality and efficiency of rRNA removal were checked with the Agilent 2100 Bioanalyser (Agilent Technologies). Libraries for RNA-Seq were then prepared using the SOLiD platform (v3.5 and 4) following manufacturer instructions (Applied Biosystems).

### Chromatin Immunoprecipitation (ChIP)

ChIP protocol and quantification was performed as previously described^65^. In brief, 3.5 × 10^7^ fixed cells were sonicated in 3 ml of SDS buffer. The lysate was diluted with 3 ml of Triton Dilution Buffer (100 mM Tris at pH 8.6, 100 mM NaCl, 5 mM EDTA, 5% Triton X-100). Immunoprecipitates were from 6 mL of dilutes lysate, with either 2 μgof polyclonal antibodies specific for total PolII (Santa Cruz, SC899) or 500 μL of blocked protein A or protein G beads (50% slurry protein A or protein G-Sepharose; Amersham). For analysis of H3K4 mono-methylation and H3K27 acetylation, we used antibodies against H3K4me1 (Abcam, ab8895) and H3K27ac (Abcam, ab4729), correspondingly. PCR was performed with 4 μL of DNA and 800 nM primers diluted in a final volume of 20 μL in SYBRGreen Reaction Mix (Perkin Elmer). Accumulation of fluorescent products was monitored by real-time PCR using a Gene-Amp 5700 Sequence Detector (Perkin Elmer). Primers for ChIP analysis are indicated in **Suppl.Table S4** (sheet 2).

### Bioinformatics and Statistics

The sequenced reads were mapped to hg19 build of the human genome from University of California Santa Cruz (UCSC) genome browser database (http://genome.ucsc.edu), using TopHat version 1.1.4 with the aligner Bowtie 0.12.7 (http://ccb.jhu.edu/software/tophat and (http://bowtie-bio.sourceforge.net)) with their default parameters except –m 1 and –segment-mismatches 2. The mapped reads were further manipulated by removing the reads that mapped to multiple locations (-q 0) using SAMtools. To create *de novo* transcriptome assembly from our RNA-Seq data sets, we used the default parameters on the Cufflinks assembler (2.0.2), which removes assembled transfrags with very low estimated abundance relative to other isoforms of the same gene. More details on these parameters can be found at the Cufflinks website (http://bio.math.berkeley.edu/cufflinks). To build our own gene model for the all the non-coding transcripts discovered we used gene assemblies from RefSeq and GENCODE (v14.0) and a long RNA-Seq transcriptome annotation of fibroblast cell-lines (BJ) provided by ENCODE Cold Spring Harbor Labs (GEO: GSE30567). Assemblies from all samples were merged using Cuffmerge and annotated as de-novo. Each transcript was labeled as RefSeq, GENCODE, ENCODE or de-novo.

Assembled Transcripts (>200 nt) from all the above annotation models (including de-novo) were grouped based on overlapping of exonic loci of gene locus on the same strand as a gene locus. RNA transcript levels were expressed as reads per kilo base per million mapped reads (RPKMs) using the Partek Genomics Suite software (Partek Inc., St.Lous, MO) and Expectation-Maximization (E/M) Algorithm (built-in Partek algorithm) was used to estimates the most likely relative RPKM levels of each assembled transcripts for a given gene locus. We then selected a representative RNA transcript for a given gene locus using as criteria their corresponding RPKM values (i.e. the transcript with the highest RPKM in any experimental condition). The selected representative transcripts were then used to re-estimate reads count and RPKM again for each transcripts and condition.

To classify stress induced non-coding transcripts, all representative non-coding transcripts were grouped based on their location to the nearest RefSeq gene (either coding or non-coding) to generate all combinations of non-coding/RefSeq gene pair.(as shown in Table 1). Finally, to generate average profiles, Average Read count Per Million mapped reads (RPM) of selected regions was calculated using ngs.plot (v2.08) (https://code.google.com/p/ngsplot/)^66^. Gene Ontology (GO) and Functional annotation (FA) analyses were performed using the DAVID bioinformatics software^67^.

### Data sets

The data in the study has been deposited into NCBI GEO under record IDs GSE55172 and GSE63112.

BJ DNaseI Hypersensitivity from ENCODE/University of Washington (GEO ID: GSM736518)^68^,^69^. BJ_H3K4me3,BJ_H3K27me3 and BJ_H3K36me3histone modifications by ChIP-seq from ENCODE/University of Washington (GEO ID:GSE35583)^68^. PolyA + CAGE RNA subcellular localization from ENCODE/RIKEN (GEO ID: GSE34448)^1^.

### *De-novo* Motif Discovery

Motif discovery was performed on −0.5 kb to −1.5 kb promoter regions upstream of the TSS of the protein-coding gene pairs of all si-paancRNAs where there was the peak of their transcriptional induction. In addition, sequences −0.5 kb to 0.5 kb around the TSS of distal lncRNAs were used to determine the enriched motifs. The enriched motifs were identified using Multiple Em For Motif Elicitations (MEME) software suite (http://meme.nbcr.net/meme/cgi-bin/meme-chip.cgi) with default settings. Reported motifs were ranked according to E-value significance, which is the estimated probability of the expected number of motifs with the given log-likelihood ratio compared to a random set of sequences of similar size and sequence width.

### Traveling Ratio

Traveling Ratio (TR) that compares the ratio between PolII density in the promoter and in the gene region was defined in previous study^70^. We defined the promoter region from −30 to +300 relative to the TSS and the gene body as the remaining length of the gene. TR values were calculated for all protein-coding partners of paancRNAs and only the annotated paancRNAs with minimum length of 600 bp.

### Reverse Transcription and quantitativePCR (RT-qPCR)

After treatment with RNase-free DNase I (Invitrogen), total RNA was reverse-transcribed, using Superscript II First-Strand Synthesis Kit for RT-PCR (Invitrogen) under the conditions defined by the supplier. cDNA was quantified by real-time PCR on the ABI Prism 7900 Sequence Detection System (Applied Biosystems) using SYBR Green PCR Supermix from Invitrogen. Each sample was run in duplicate and each PCR experiment included two non-template control wells. Expressions were normalized to β-actin (ACTB) or glyceraldehyde-3-phosphate dehydrogenase (GAPDH). Primers used for qPCR analysis are indicated in **Suppl.Table S4** (sheet 1).

### Polysome Fractionation

Polysome fractionation was carried as described earlier^71^. Briefly, cells were incubated with 150 μg/ml of cycloheximide (Sigma-Aldrich) for 15 min prior to harvesting. Approximately 1 × 10^7^ cells were lysed in 500 μl of lysis buffer [5 mM Tris-HCl, 2.5 mM MgCl_2_, 1.5 mM KCl, 0.5% Triton X, 0.5% Sodium deoxycholate, 2 mM DTT, 40 U/μl of RNasin (Fermentas) and 1 mM DTT]. Then cell lysates were subjected to centrifugation for 3 min at 14,000 rpm at 4 °C to obtain clarified lysate. Absorbance at 260 nm was measured and 16-optical density (A260) units of the lysate in 300 μl lysis buffer were layered onto 4-ml 5-45% linear sucrose gradients made in a 20 mM HEPES buffer, containing 100 mM KCl, 5 mM MgCl2, 0.5mg/ml heparin, 100 μg/ml cycloheximide. Centrifugation was carried out at 130,000 x g in a SW40-Ti swinging bucket rotor (Beckman) for 1.5 hrs at 4 °C. Eight fractions were collected and used for RNA isolation. Polysome profiles were monitored at 254 nm using an ISCO UA-6 UV detector. The assignment of OD254 peaks corresponding to the 40S and 60S subunits and to intact ribosomes was confirmed with the Agilent 2100 Bioanalyzer. The positions of higher ribosomal oligomers were estimated by extrapolation of a curve fit to these points. Each fraction was adjusted to 0.5% SDS, and the 8 fractions were combined to form four pools. Fractions 1-4 and 5-8 were combined as pools 1 (Untranslated) and 2 (Translated), respectively (Fig. 6d). RNA isolated from each pool was used for further analysis. In parallel, total RNA was also isolated from unfractionated lysates for transcriptional analysis.

RNA Isolation from Polysome Fractions: Polysomal fractions were spiked with bacterial spike-in (Affymetrix) prior to precipitation, which served as isolation controls. RNA was precipitated at −70 ^0^C with 2.5 vol of 100% ethanol and 1/10th vol of 10 mM LiCl. RNA was extracted from precipitated fractions using Qiagen RNeasy mini-columns.

For microarray analysis of polysome fractions MRC5 cell lysates were prepared as described above. The lysates were fractionated by centrifugation in 15-50% linear sucrose gradients at 36,000 g in SW41-Ti rotor (Beckman) for two hours at 8 ^0^C. Twenty-three fractions were collected using Piston Gradient Fractionator (Biocomp) and RNA was isolated from the fractions using phenol chloroform extraction. Briefly, RNA was extracted two times in equal volume of 25:24:1 phenol:chloroform:isoamyl alcohol followed by extraction in an equal volume of 49:1 chloroform:isoamyl alcohol. RNA was precipitated at −80 °C with 2.5 volumes of 100% ethanol and 1/10 th volume of sodium acetate (3M), pH5.2.

### Custom microarray

Custom microarray was designed based on Agilent platform, format 8 × 60 K (Design ID 047718). The probes were designed against Gencode v15 database. Two probes targeted each of 22,001 lncRNA transcripts and 17,535 randomly selected protein coding targets. Samples from 23 polysome fractions were divided into 3 pools: untranslated (fractions 1-9,representing free RNPs and 80S ribosomes); low-translated (fractions 10-14, representing 2-4 ribosomes) and highly-translated (fractions 15-23 representing 5 and more ribosomes). The procedure was carried out three times, each with an independent RNA preparation and each analyzed with 3 microarrays, both in control and OS condition (2 hours) together with whole-cell RNA for each condition. In brief, 100 ng of total RNA from whole cell extracts and polysome fractions was labeled using LowInputQuick Amp Labeling kit (Agilent 5190-2305) following manufacturer instructions. Briefly, mRNA was reverse transcribed in the presence of T7-oligo-dT primer to produce cDNA. cDNA was then *in vitro* transcribed with T7 RNA polymerase in the presence of Cy3-CTP to produce labeled cRNA. The labeled cRNA was hybridized to the Agilent SurePrint G3 gene expression 8 × 60 K microarray according to the manufacturer’s protocol. The arrays were washed, and scanned on an Agilent G2565CA microarray scanner at 100% PMT and 3μm resolution. Intensity data was extracted using the Feature Extraction software (Agilent). Raw data was taken from the Feature Extraction output files and was corrected for background noise using the normexp method^72^. To assure comparability across samples we used quantile normalization. All statistical analyses were performed with the Bioconductor project (http://www.bioconductor.org/) in the R statistical environment (http://cran.r-project.org/)^73^.

The 24 Agilent features extraction results text files of both, stress and control in triplicates (as mentioned in the Sample section) were imported into Partek Genomics Suite. The gProcessed Signal parameter was imported into the Partek probe intensity spreadsheet and the data was further log2 transformed. The imported probe intensity log2 transformed data was separated into two categories: Coding and Non-coding. For both of these categories the following steps were done separately: 1) Normalization and Quality Control; the data was subjected to quantile normalization and quality control check by hierarchical clustering^74^. The anomalous replicates that failed to cluster with the other replicates were removed and quantile normalization was redone on the remaining samples. 2) Computation of Differential Expression; an Analysis of variance (ANOVA) containing contrasts for Stress versus Control for the whole-cell (Total) RNA, None, Low and High fractions was performed to obtain the ratios and fold change of Stress over Control for the Total and the Fractionated RNA as follows: RT = ST vs CT, RN = SN vs CN, RL = SL vs CL and RH = SH vs CH(R = Ratio, S = Stress, C = Control, T = Total, N = None, L = Low fraction and H = High fraction). 3) Detection of Transcriptional Regulation; to obtain the transcriptionally regulated probes, the list of significant probes satisfying the p-value criteria (p-value < 0.1) for Stress versus Control samples in Total RNA was classified into three groups using a fold change cut-off of 1.5:Transcriptionally High (> = 1.5), Transcriptionally Low (= < −1.5) and Transcriptionally No change (>−1.5 and <1.5). 4) Detection of Translational Regulation; for each of the significant probes in the three transcriptionally classified groups, the ratios of Stress over Control for the Fractionated RNA (None, Low, High) were compared and the absolute difference was calculated as follows:D1 = abs(RN-RL), D2 = abs(RL-RH), D3 = abs(RN-RH). If any of these absolute differences were greater than 1 (D1 > = 1 or D2 > = 1 or D3 > = 1), it was considered to be showing a shift in translation or in other words being translationally regulated. The ratio of Stress over Control for the fractionated RNA (RN/RL/RH) of these translationally regulated probes were compared to obtain the one having the highest value. A count of the highest ratio was kept to determine the trend for each transcriptionally classified group and finally for the Coding and Non-Coding categories on the whole.

## Additional Information

**How to cite this article**: Giannakakis, A. *et al.* Contrasting expression patterns of coding and noncoding parts of the human genome upon oxidative stress. *Sci. Rep.* **5**, 9737; doi: 10.1038/srep09737 (2015).

## Supplementary Material

Supporting Information

Supporting Information

Supporting Information

Supporting Information

Supporting Information
