# Supplementary material for: Contrasting expression patterns of coding and noncoding parts of the human genome upon oxidative stress
Source: Sci Rep. 2015 May 29;5:9737. doi: 10.1038/srep09737 (PMC4448690; doi:10.1038/srep09737)

Contrasting expression patterns of coding and noncoding parts of the human genome upon oxidative stress

Antonis Giannakakis[1], Jingxian Zhang[2,3+], Piroon Jenjaroenpun[1+], Srikanth Nama[3,4], Norliyana Zainolabidin[2,3], Mei Yee Aau[2], Aliaksandr A. Yarmishyn[1], Candida Vaz[1], Anna V. Ivshina[1], Oleg V. Grinchuk[1], Mathijs Voorhoeve[3,5], Leah A. Vardy[4,6], Prabha Sampath[4,3], Vladimir A. Kuznetsov[1,7*], Igor V. Kurochkin[1*] and Ernesto Guccione[2,3*]

SUPPLEMENTARY FIGURES

Figure S1. Analysis of the cytosolic transcriptional-response of fibroblast cell lines upon OS**.**

Figure S2. Box-plot graphs of the length distribution of annotated/de-novo predicted paancRNAs (**a**) dncRNAs (**b**) and terminal-associated concurrent (**c**).

Figure S3. a. Chip qPCR validation of the PolII accumulation at si-paancRNA loci after 30 minutes and 2 hours of OS in MRC5 cells **b.** PolII traveling ratio distribution of RefSeq si-paancRNAs and the coding partners of all si-paancRNAs at 30 minutes and 2 hours of OS. Higher TR (Traveling Ratio) values indicate higher degree of pausing.

Figure S4. De-novo motif analysis performed on the genomic region (500 bp) around the TSS of the annotated dncRNAs (N=1543) generated three statistically significant motives (motif1 present on 1000 times/1543 dncRNAs=64.8%, motif2: 659times/1543dncRNAs =42.7%, motif3: 895times/1543dncRNAs =58.0%).

Figure S5. a. Venn diagram of all RefSeq paancRNAs induced more than 1.5-fold change in all time-points and both cell lines compared to untreated cells **B.** RT-qPCR validation of the up-regulation of all common si-paancRNAs in all time-points and cell lines.

Figure S6. a. Kinetic studies of RefSeq paancRNAs transcript levels in OS alone (red line) or followed by removal of the stimulus, H_2O_2 (blue line) or after 1 hour pre-treament with ActD (green line) together with their coding partners **b.** ROS scavenging, by N-acetylcysteine pre-

treatment (NAC, 1mM and 5mM, 1hour), followed by 2 hours of H_2O_2 treatment, prevented upregulation of paancRNAs.

Figure S7. Oxidative stress-induced distal lncRNAs (si-dncRNAs) show bi-directional transcription and polII distribution similar to protein-coding genes. **a.** dncRNAs are up-regulated in response to OS and show, at a subset of their promoters, upstream antisense transcription. Overlap between stress-induced dncRNAs and **b.** DNase I hypersensitivity sites. **c.** CAGE transcripts **d.** histone marks **e**. RT-qPCR validation of five RefSeq dncRNAs that were found to be commonly up-regulated (>1.5-Fc) in both cell lines and at both time-points upon OS. **f.** Overlap between stress-induced dncRNAs and PolII (TSS and TES of dncRNAs is indicated on the x-axis. **g.** Examples of PolII binding and RNA-seq read distribution at stress-induced dncRNAs loci.

Figure S8. a. Bar graph of the coding bias index for each si-paancRNA together with controls (MALAT/NEAT/18S/ACTB/GAPDH/FOS) used for the polysome profiling analysis coupled to RNA isolation and RT-qPCR**b.** Polysome profiling of total RNA from untreated and treated (0.2mM H_2O_2 for 2 hrs) MRC5 cells **c.** Table showing the number of promoter-associated, terminal-associated and distal lncRNAs that was present on the applied custom microarray. Among all three lncRNAs classes, si-paancRNAs (promoter-associated opposite) showed the highest number of transcripts being up-regulated in OS and associating with polysomes. **d.** qRT-PCR analysis of *NR_036539* si-paancRNA (example of a lncRNA which associates with polysomes), *GAPDH*(example of a coding mRNA which moves away from polysomes) and *ATF3*(representative example of a coding mRNA with increased association with polysomes). Individual fractions were obtained from sucrose density gradient of cytoplasmic lysates of untreated (blue line) and treated with 0.2mM H_2O_2 for 2 hrs (red line) MRC5 cells.

Figure S1

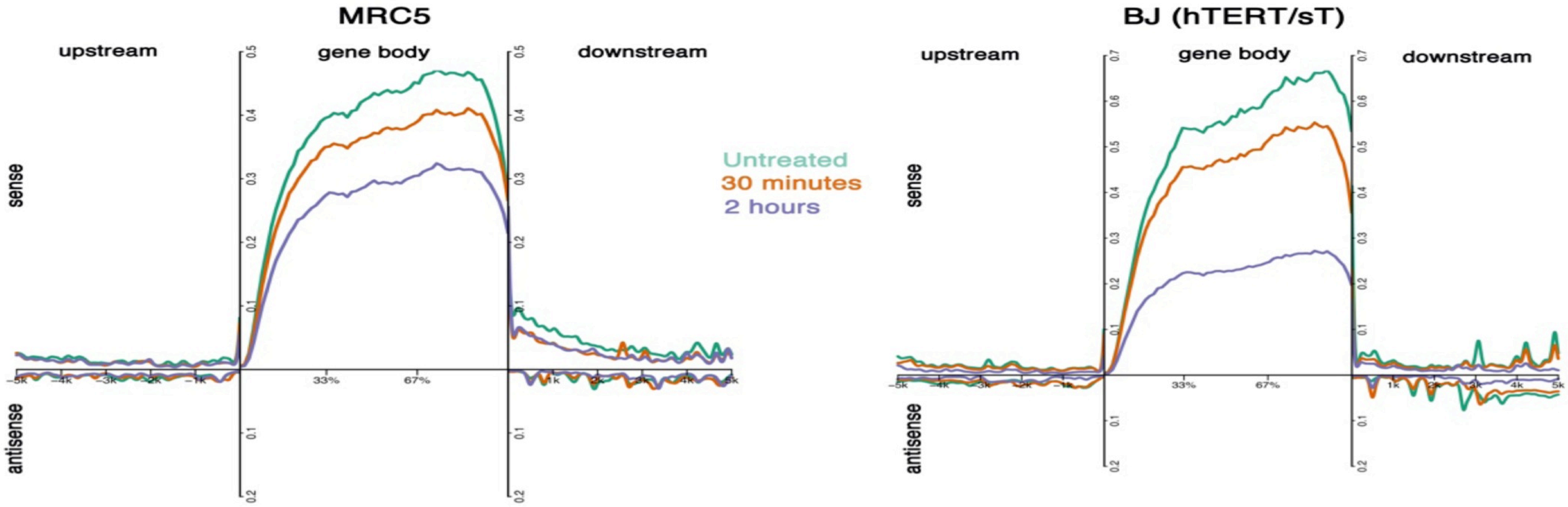
MRC5
upstream
gene body
downstream
sense
antisense
Untreated
30 minutes
2 hours
33%
67%
BJ (hTERT/sT)
upstream
gene body
downstream
sense
antisense

Figure S2

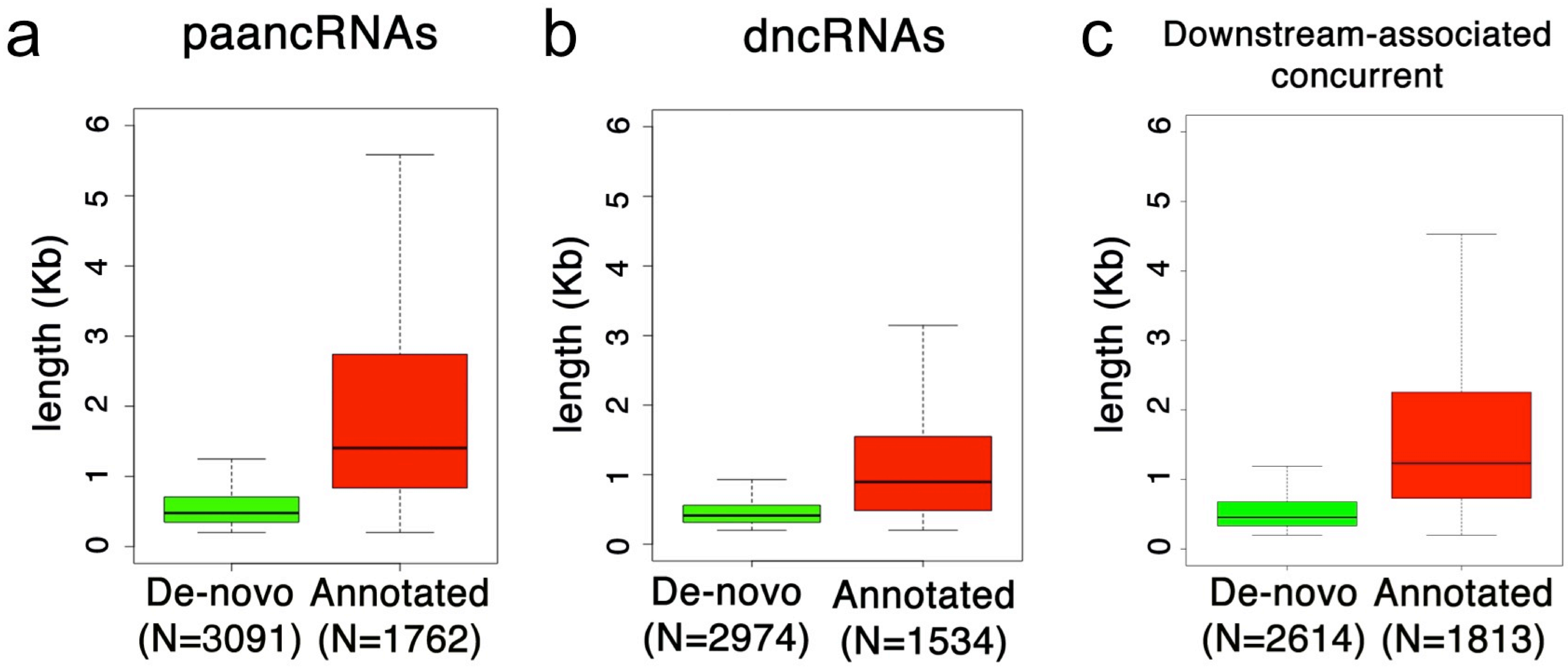
a
paancRNAs
b
dncRNAs
c
Downstream-associated concurrent
length (Kb)
0
1
2
3
4
5
6
De-novo (N=3091)
Annotated (N=1762)
De-novo (N=2974)
Annotated (N=1534)
De-novo (N=2614)
Annotated (N=1813)

Figure S3

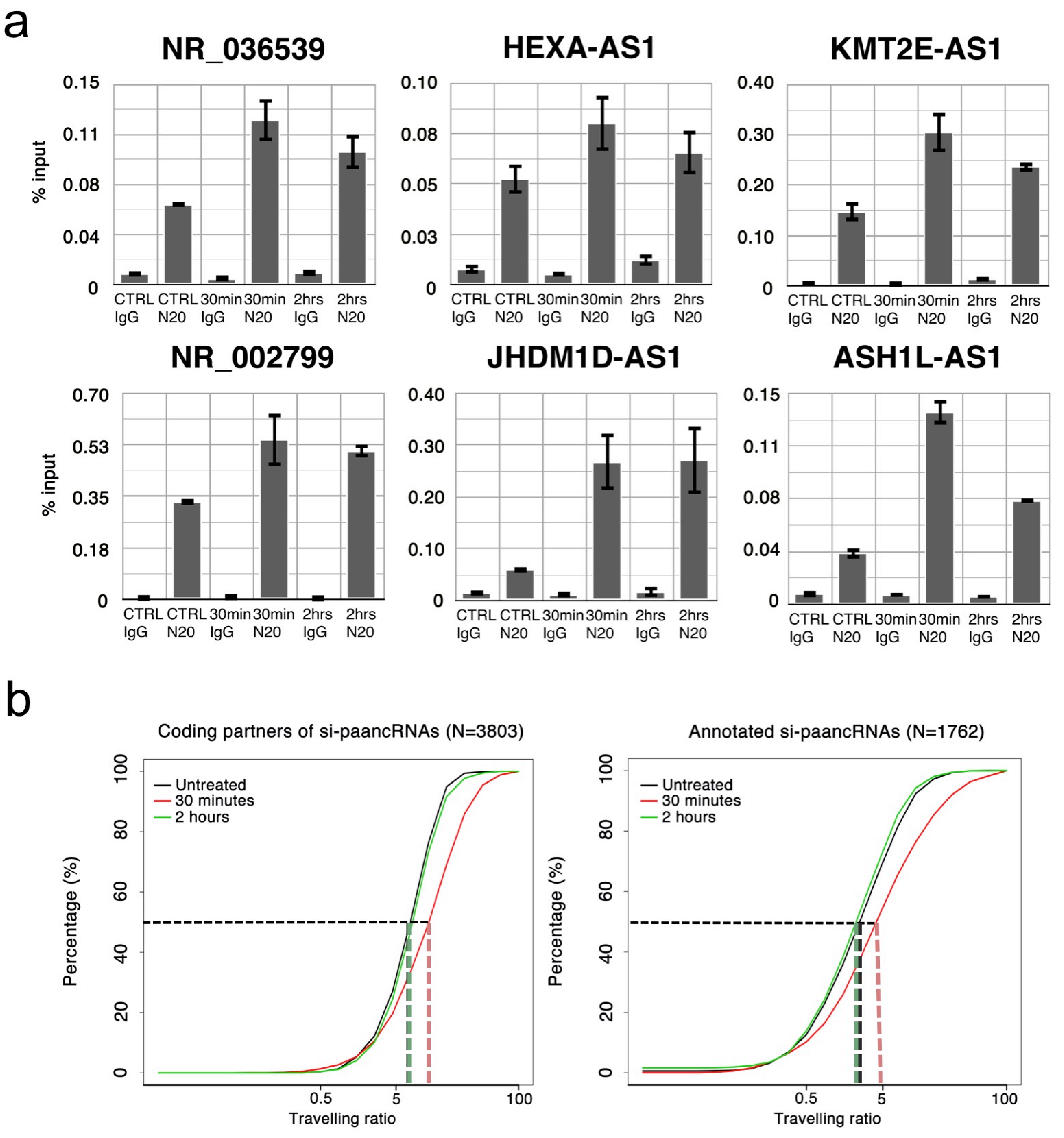
a
NR_036539
HEXA-AS1
KMT2E-AS1
NR_002799
JHDM1D-AS1
ASH1L-AS1
% input
CTRL IgG
CTRL N20
30min IgG
30min N20
2hrs IgG
2hrs N20
b
Coding partners of si-paancRNAs (N=3803)
Annotated si-paancRNAs (N=1762)
Untreated
30 minutes
2 hours
Percentage (%)
Travelling ratio

Figure S4

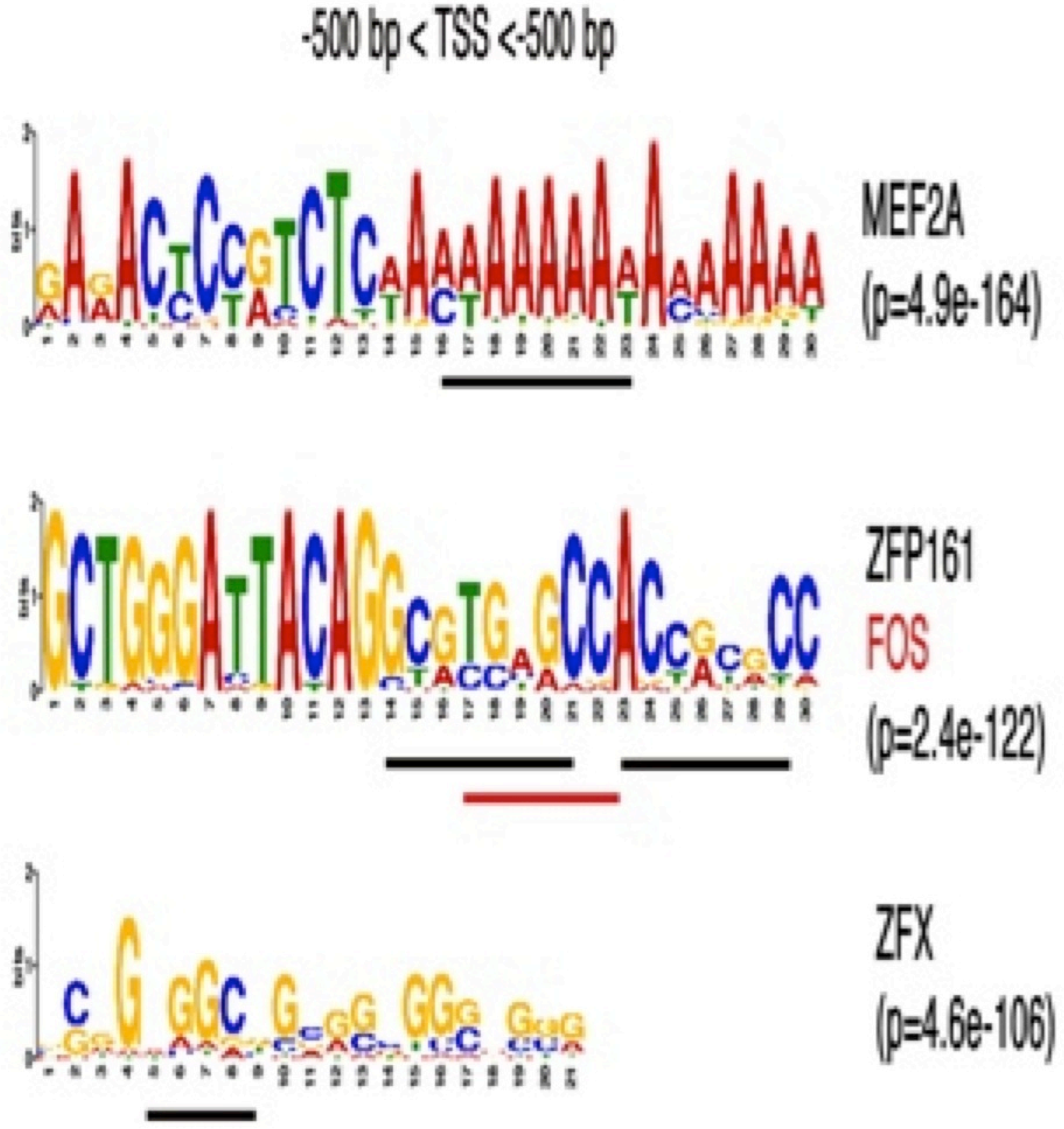

-500 bp < TSS <-500 bp
MEF2A
(p=4.9e-164)
ZFP161
FOS
(p=2.4e-122)
ZFX
(p=4.6e-106)

Figure S5

a

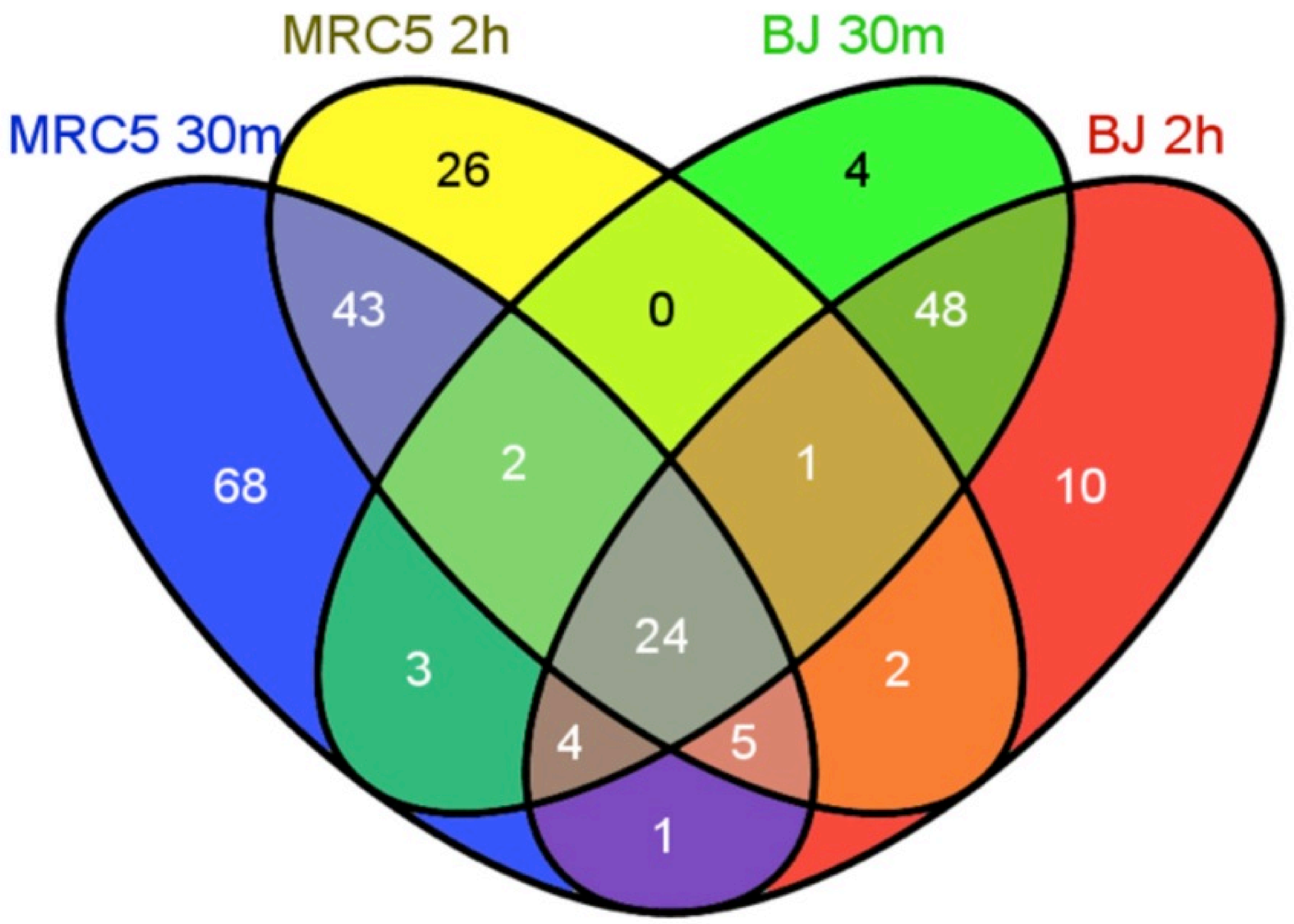

MRC5 2h
BJ 30m
MRC5 30m
BJ 2h
26
4
43
0
48
68
2
1
10
24
3
2
4
5
1

b

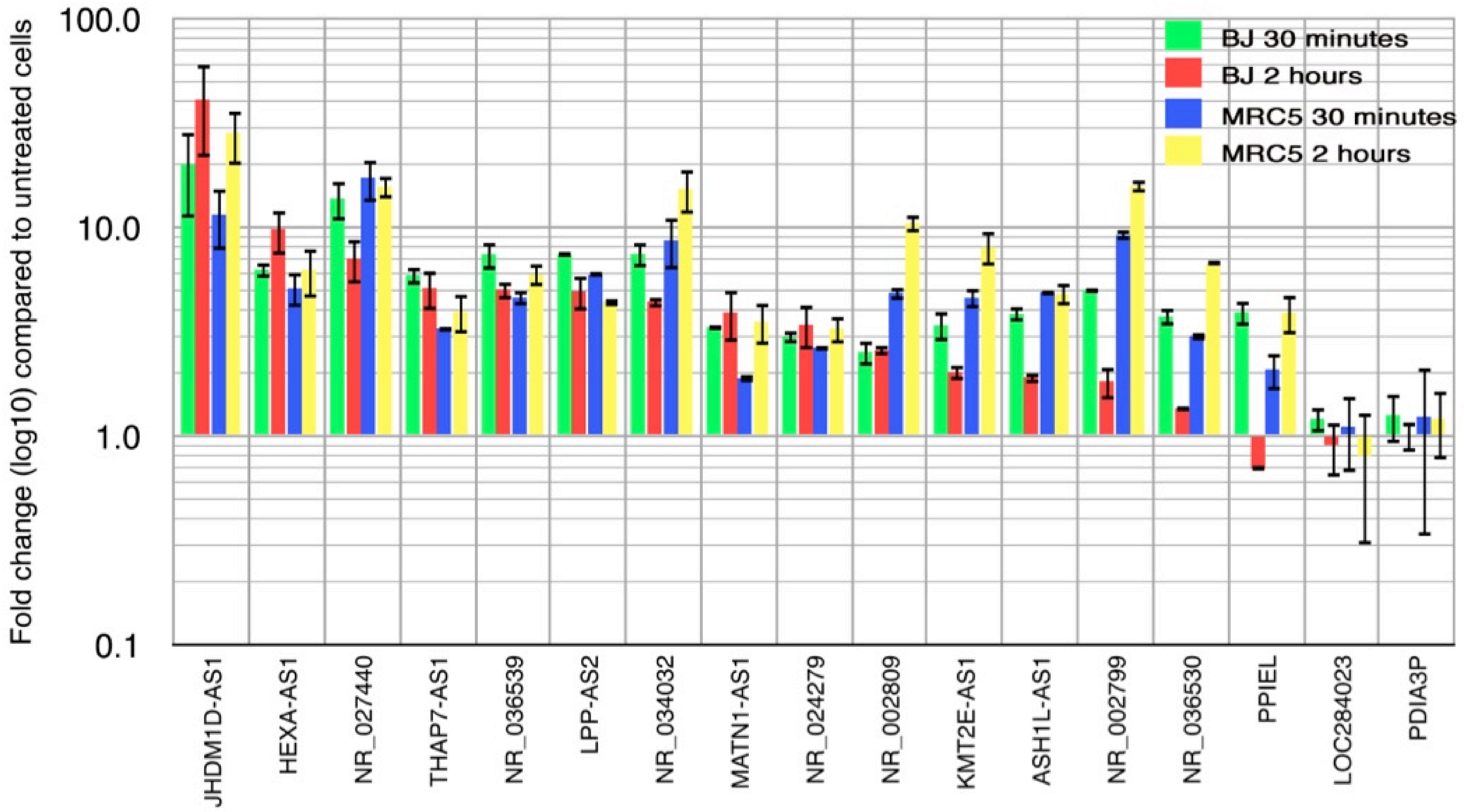

Fold change (log10) compared to untreated cells
100.0
10.0
1.0
0.1
BJ 30 minutes
BJ 2 hours
MRC5 30 minutes
MRC5 2 hours
JHDM1D-AS1
HEXA-AS1
NR_027440
THAP7-AS1
NR_036539
LPP-AS2
NR_034032
MATN1-AS1
NR_024279
NR_002809
KMT2E-AS1
ASH1L-AS1
NR_002799
NR_036530
PPIEL
LOC284023
PDIA3P

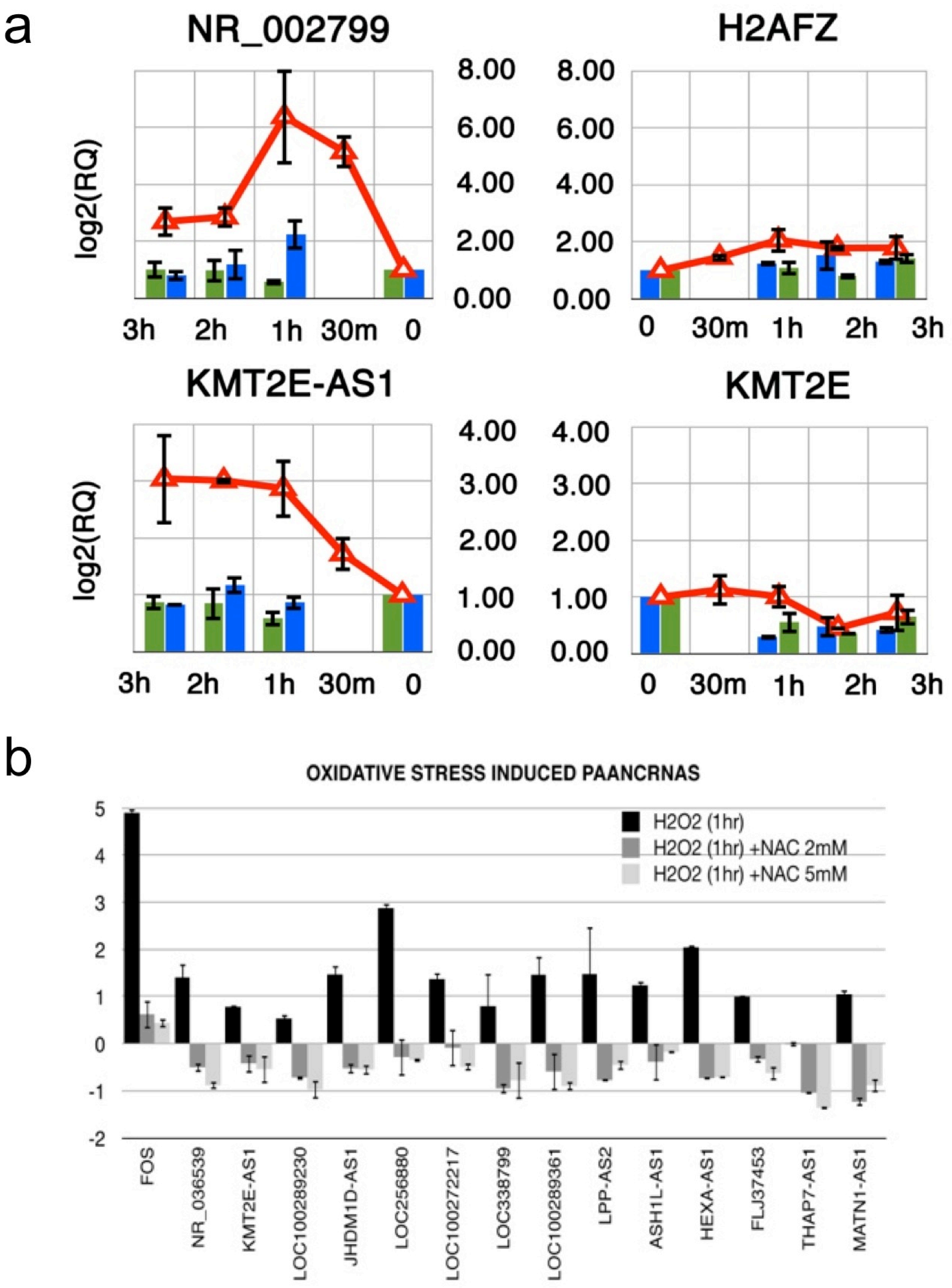
a
NR_002799
H2AFZ
log2(RQ)
8.00
6.00
4.00
2.00
0.00
3h 2h 1h 30m 0
0 30m 1h 2h 3h
KMT2E-AS1
KMT2E
4.00
3.00
2.00
1.00
0.00
b
OXIDATIVE STRESS INDUCED PAANCRNAS
H2O2 (1hr)
H2O2 (1hr) +NAC 2mM
H2O2 (1hr) +NAC 5mM
5
4
3
2
1
0
-1
-2
FOS
NR_036539
KMT2E-AS1
LOC100289230
JHDM1D-AS1
LOC256880
LOC100272217
LOC338799
LOC100289361
LPP-AS2
ASH1L-AS1
HEXA-AS1
FLJ37453
THAP7-AS1
MATN1-AS1

Figure S7

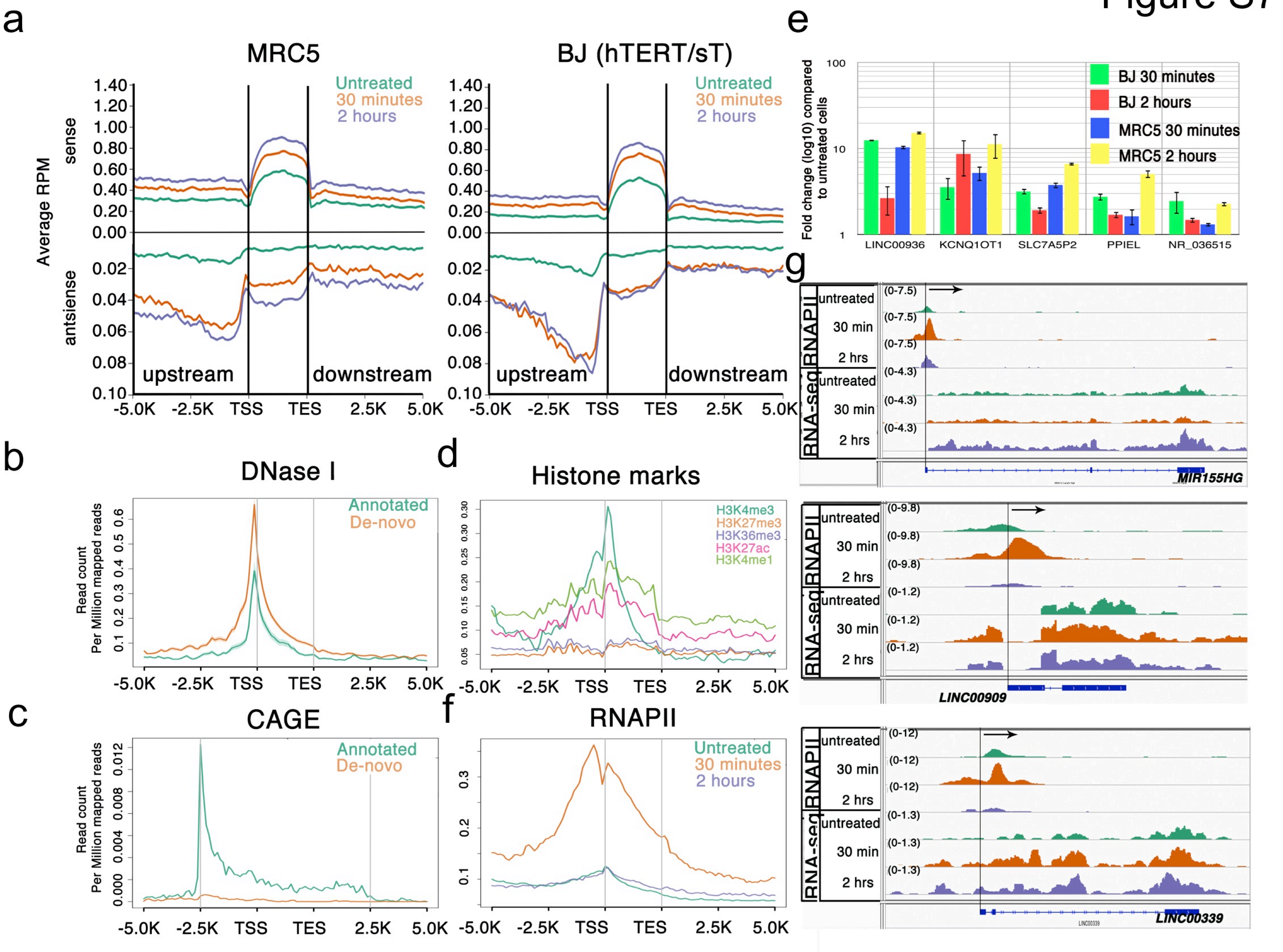

a
MRC5
BJ (hTERT/sT)
Untreated
30 minutes
2 hours
Average RPM
sense
antisense
upstream
downstream
-5.0K
-2.5K
TSS
TES
2.5K
5.0K
b
DNase I
Annotated
De-novo
Read count Per Million mapped reads
c
CAGE
d
Histone marks
H3K4me3
H3K27me3
H3K36me3
H3K27ac
H3K4me1
f
RNAPII
e
Fold change (log10) compared to untreated cells
BJ 30 minutes
BJ 2 hours
MRC5 30 minutes
MRC5 2 hours
LINC00936
KCNQ1OT1
SLC7A5P2
PPIEL
NR_036515
g
RNAPII
RNA-seq
untreated
30 min
2 hrs
MIR155HG
LINC00909
LINC00339

Figure S8

a

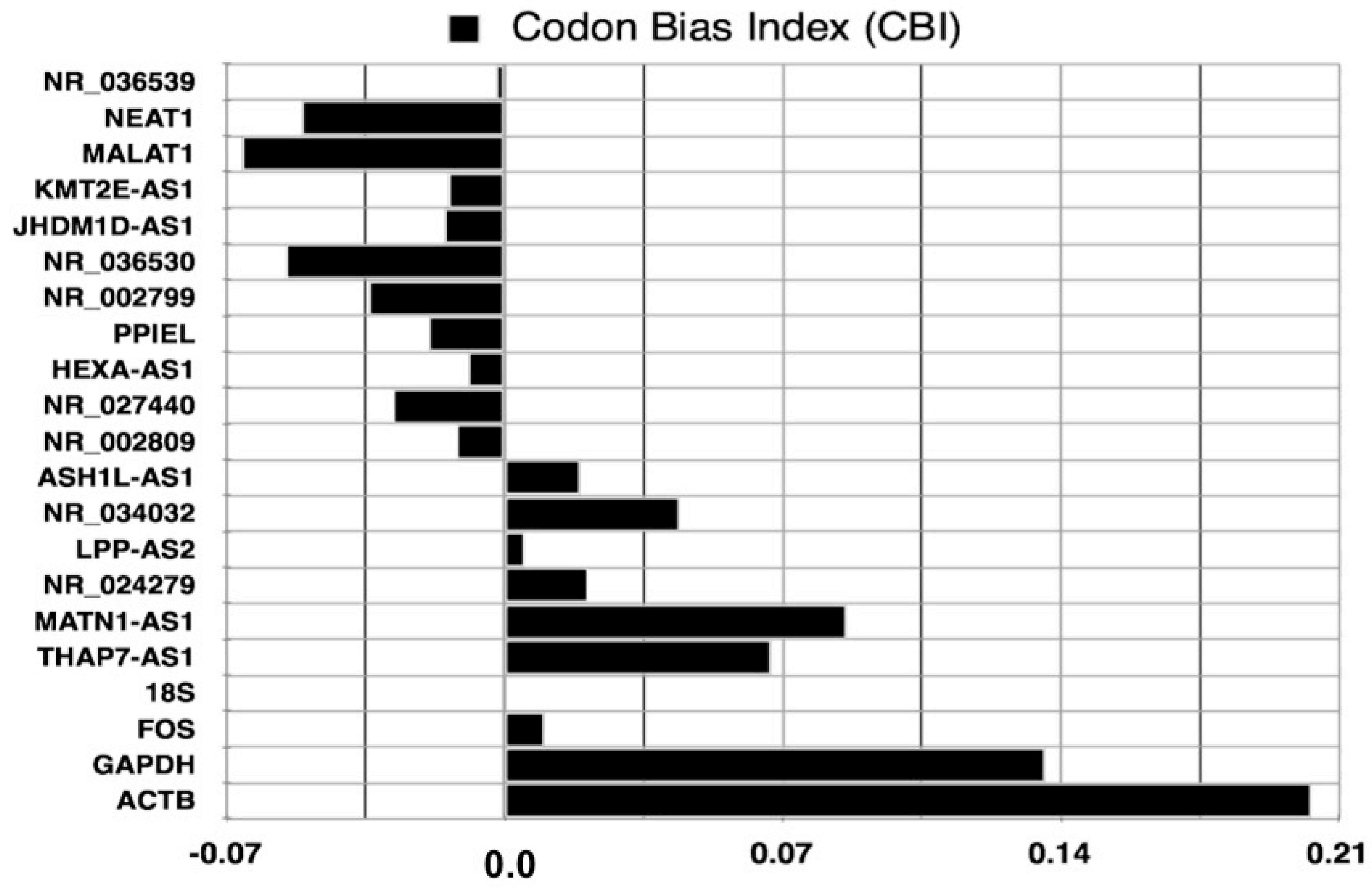

b

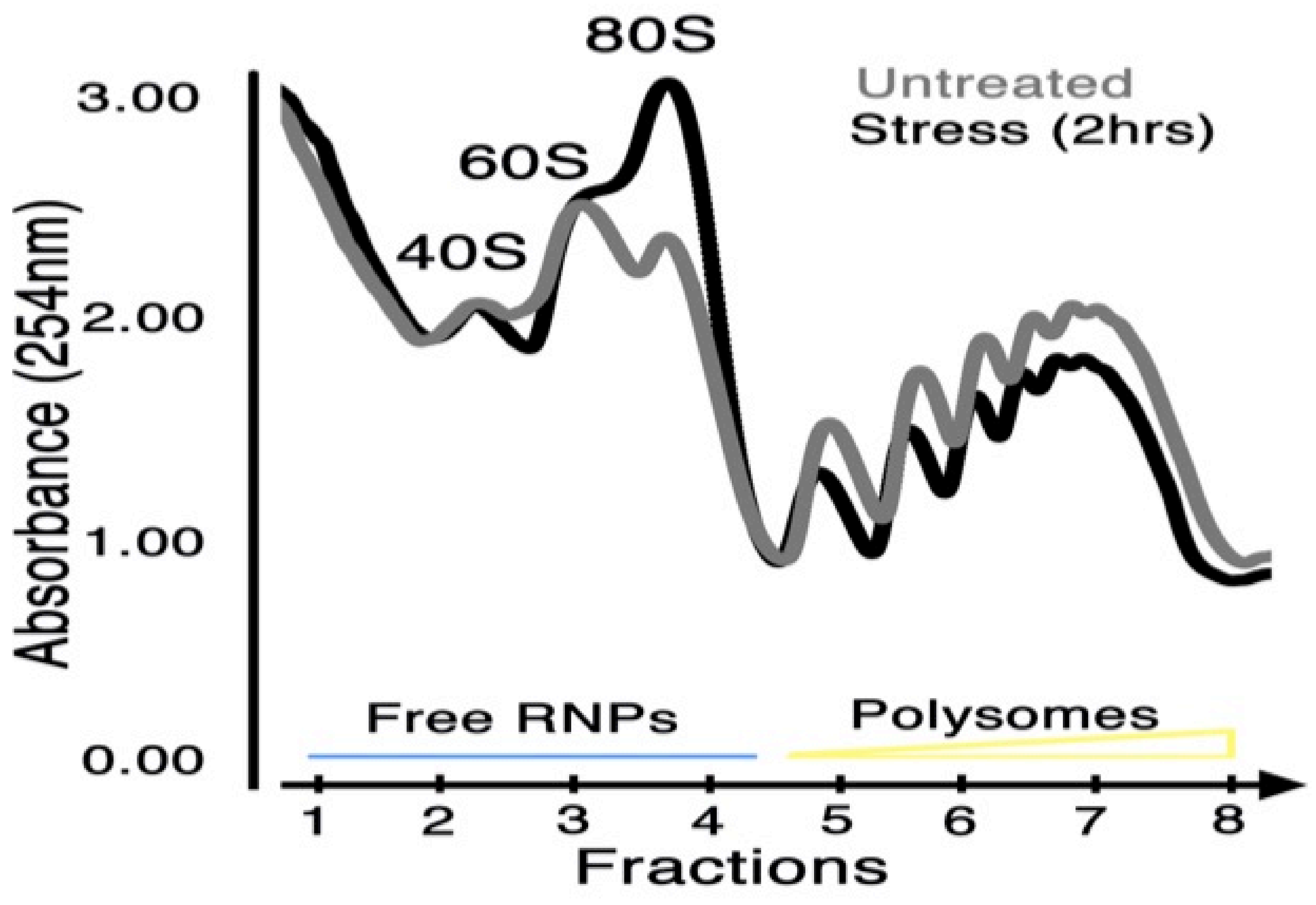

c

	Classification	No polysomes		Light polysomes		Heavy polysomes	
		concurrent	opposite	concurrent	opposite	concurrent	opposite
OS-induced lncRNAs	promoter -associated	33	136	38	189	43	322
	downstream -associated	23	73	43	89	64	133
	distal	51	45	60	50	71	62

d

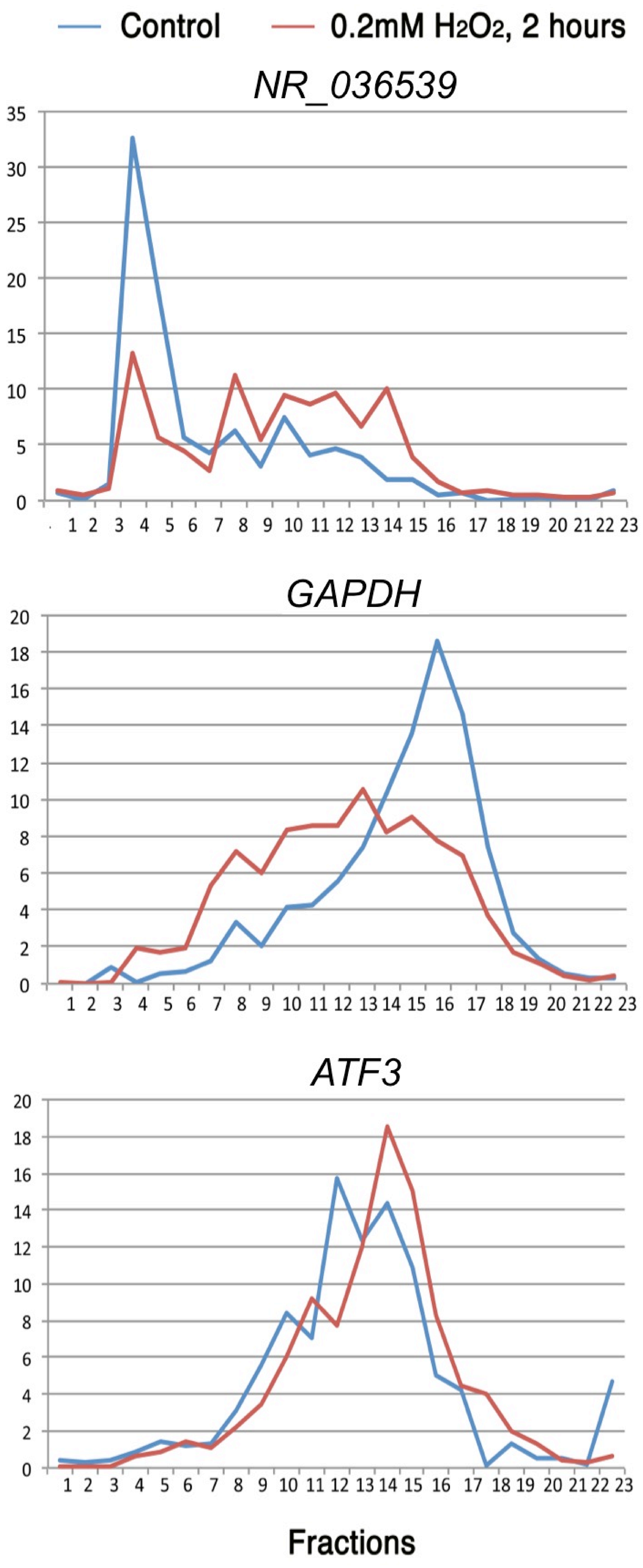

Supplement: Supporting Information [file srep09737-s1.pdf]
